# Proteomic and metabolomic approach to rationalize the differential mosquito larvicidal toxicity in *Bacillus* sp. isolated from the mid‐gut of *Culex quinquefasciatus* mosquito larvae

**DOI:** 10.1002/ansa.202000081

**Published:** 2020-10-12

**Authors:** Domnic Colvin, Vishnu Dhuri, Mahesh Samant, Hirday Verma, Rama Lokhande, Avinash Kale

**Affiliations:** ^1^ School of Chemical Sciences UM‐DAE Centre for Excellence in Basic Sciences University of Mumbai Mumbai India; ^2^ National Centre for Nanosciences and Nanotechnology University of Mumbai Mumbai India; ^3^ Department of Lifesciences Jaipur National University Jaipur India

**Keywords:** *Culex quinquefasciatus*, larvicidal toxins, mass spectrometry, metabolomics, mosquitos, proteomics, scanning electron microscopy

## Abstract

From the distinct wild locations of the Mumbai (India), dead Culex mosquito larvae were collected. The mid‐gut micro‐flora of these dead mosquito larvae was isolated on three different media that were selective for only the Gram‐positive bacteria. These bacteria were tested against the third instar stage of *Culex quinquefasciatus *larvae, cultured in the laboratory, for their larvicidal activity. After performing the toxicity assay four times in duplicates, the average statistical values showed four bacteria exhibiting differential toxicities. Identification of these strains was done by 16S rRNA sequencing and their respective surface morphologies were studied by scanning electron microscopy (SEM). The differential toxicities of the four identified Bacillus strains were rationalized by performing differential proteomics and metabolomics approach using LC‐MS and these results were analyzed against customized mosquito larvicidal toxin database which was further compared with the in silico p‐BLAST data of that respective Bacillus sp. from the NCBI database. The presence and significance of the various mosquitocidal toxins in the identified Bacillus sp. are elucidated. The present study also attempted to identify new bacterial species exhibiting mosquitocidal toxicities that have not been reported earlier.

## 1 INTRODUCTION

The mosquito‐borne diseases are one of the major reasons for high human mortality and morbidity rate, especially in tropical regions across the globe. These include malaria, dengue, yellow fever, filariasis, and chikungunya.^1^ Every year, globally more than 0.4 million deaths happen of the people because of malaria and out of which most of them are children under the age of 5 years. More than 3.9 billion people in over 128 countries are at the risk of contracting dengue, with 96 million cases estimated per year.^2^ Since 2014, major outbreaks of mosquito‐borne diseases like dengue, malaria, chikungunya, yellow fever, and zika have affected large populations across the globe and have many claimed lives, thereby putting excessive pressure on the health systems in many countries. All of these dangerous human diseases are transmitted by different species of mosquitoes amongst humans or from animals to humans. Mosquitoes are blood‐sucking insects that ingest the disease‐producing micro‐organisms during their blood meal from the infected host (animal or human) and later inject it in the new host during their subsequent new blood meal.^2^ More the number of mosquitoes in an environment, the maximum is the risk of spread of the diseases which eventually affect the health and well‐being of millions of people thereby causing an impediment to the social and economic development.^3^

Insecticides have always played a vital role in the prevention and control of mosquito‐borne diseases. Since public health pesticides are used near residential areas of the humans and also in sensitive ecological areas, their proper management is very essential.^3^ In the malaria control program, maximum reliance was on DDT for the residual spray but currently different vector management strategies are adopted. These include insecticides (DDT, Malathion, and Synthetic Pyrethroids) for residual spray; insecticide‐treated nets or Long‐Lasting Insecticidal Nets (LLIN) in high‐risk areas; larvicides mainly in urban and peri‐urban areas; biological control through larvivorous fish; elimination of vector breeding sources and sanitation.^4^ There is ample evidence that no single method is likely to provide a solution in all situations.^5^ The recurring use of man‐made insecticides for mosquito control upsets the natural biological control organization and lead to re‐emergence of mosquito populations.^6^

Microbial larvicides are bacteria that are known to control the population of mosquito larvae and hence can be used as pesticides. They serve as the best substitute for chemical larvicides by being biodegradable and environment friendly. The most commonly used entomo‐pathogenic bacteria are *Bacillus thuringiensis *subspecies *israelensis *and *Bacillus sphaericus*. As there have been reports of mosquitos developing resistance against the commonly used chemical pesticide, these bacteria have gained utmost importance. Moreover, these chemical pesticides have severe effects on humans and the ecosystem worldwide.^7^ However, recent reports have shown that even for these microbial larvicides mosquitos have developed resistance in some areas of the world.^8^, ^9^ Therefore, the resistance of mosquito populations to these* Bacillus sp*. toxins would seriously threaten the sustainability of current mosquito control programs, for using them as a microbial insecticide. Though, *B. sphaericus and B. thuringiensis *are quite effective in controlling mosquito population, the recent development of resistance in *Culex *species has severely hampered the progress in mosquito control operations.^10^, ^11^, ^12^, ^13^

Thus, screening for novel bacterial strains having similar mosquitocidal spore/crystal toxins is required to counter the resistance development shown by the current population of the mosquitos. To achieve this we collected collect dead mosquito larvae of *Culex* sp. from different locations of Mumbai, India, and to isolate the gram‐positive bacteria from their mid‐gut micro‐flora. These isolated bacteria were the tested against the third instar stage of *C. quinquefasciatus* mosquito larvae, cultured in the laboratory, for their toxicities. 16S rRNA sequencing was used to identify the four *Bacillus* strains, exhibiting the differential larvicidal activity. The surface morphologies for these bacteria were studied using Scanning Electron Microscopy (SEM). To the best of our knowledge we are the first ones to report, in our present work, the larvicidal activity of *Bacillus paramycoides*, *Bacillus australimaris, Bacillus cereus*, and *Bacillus tequilensis* to be 55%, 40%, 35%, and 30% respectively. We had used differential proteomics and metabolomics approach to rationalize these distinct toxicities.

The usage of LCMS in high throughput proteomics and metabolomics has helped us to generate big large scale data sets, which are helpful in identifying novel toxicity markers/candidates against mosquito larvae. In our study, we report the usage proteomics and metabolomics approach in our identified Bacillus sp. for the identification of potential larvicidal components in them. We also report our *in silico* analysis to further strengthen our findings from our proteomic data.

## 2 MATERIALS AND METHODS

All the reagents used in the study were from Sigma–Aldrich or else mentioned in the respective protocols.^14^, ^15^, ^16^, ^17^, ^18^, ^19^, ^20^, ^21^, ^22^, ^23^, ^24^, ^25^, ^26^, ^27^

### 2.1 Collection and midgut dissection of mosquito larvae

The detailed protocol is available on protocols.io.

https://doi.org/10.17504/protocols.io.bjdhki36

### 2.2 Toxicity assay for mosquito larvicidal activity

The detailed protocol is available on protocols.io.

https://doi.org/10.17504/protocols.io.bjdiki4e

### 2.3 AND

https://doi.org/10.17504/protocols.io.bmjrk4m6

### 2.4 DNA extraction and Sequencing

The detailed protocol is available on protocols.io.

https://doi.org/10.17504/protocols.io.bjdxki7n

### 2.5 SEM imaging of Bacteria

The detailed protocol is available on protocols.io.

https://doi.org/10.17504/protocols.io.bjdjki4n

### 2.6 In silico analysis

The detailed protocol is available on protocols.io.

https://doi.org/10.17504/protocols.io.bjdjki4n

### 2.7 Protein Extraction

The detailed protocol is available on protocols.io.

https://doi.org/10.17504/protocols.io.bjdnki5e

### 2.8 Trypsin digestion

The detailed protocol is available on protocols.io.

https://doi.org/10.17504/protocols.io.bjdpki5n

### 2.9 De‐salting of tryptic digested peptides

The detailed protocol is available on protocols.io.

https://doi.org/10.17504/protocols.io.bjdqki5w

### 2.10 Metabolite Extraction

The detailed protocol is available on protocols.io.

https://doi.org/10.17504/protocols.io.bjdtki6n

### 2.11 LC‐MS/MS analysis

The detailed protocol is available on protocols.io.

https://doi.org/10.17504/protocols.io.bjdvki66

### 2.12 Database preparation and search for protein identification

The detailed protocol is available on protocols.io.

https://doi.org/10.17504/protocols.io.bjdwki7e

## 3 RESULTS

### 3.1 Identification of the bacteria exhibiting toxicity against *Culex quinquefasciatus*

There were four different bacterial strains obtained in the study which showed differential toxicities against the third instar stage mosquito larvae of *Culex quinquefasciatus*. Based on their morphological tests these four bacteria were found to be rod‐shaped and gram‐positive Bacillus sp. These bacteria were initially sequenced for identification using three primers and then further confirmed by re‐sequencing them using five different primers for their 16S rRNA. On comparing with NCBI database the 16S rRNA sequences thus obtained were identified as *Bacillus paramycoides* strain MCCC 1A04098 (Max score: 1541, Identity: 97% and Query covered: 93%) which showed maximum toxicity of 55%. The second most toxic was 40% identified as *Bacillus australimaris* strain MCCC 1A05787 (Max score: 1757, Identity: 99% and Query covered: 99%). The median toxicity of 35% was shown by *Bacillus cereus* CCM 2010 (Max score: 2789, Identity: 99% and Query covered: 99%), and the least toxic strain with only 30% toxicity was identified as *Bacillus tequilensis* 10b (Max score: 2501, Identity: 99% and Query covered: 96%) as shown in Table 1.

**TABLE 1:** Identification of the *Bacillus* species using 16S rRNA sequencing and comparing against NCBI database

	Toxicity	Description	Strain	Max score	Total score	Query cover	E‐ value	Identity
Strain no.1	55%	*Bacillus paramycoides*	MCCC 1A04098	1541	3073	93%	0.0	97%
Strain no. 2	40%	*Bacillus australimaris*	MCCC 1A05787	1757	3488	99%	0.0	99%
Strain no. 3	35%	*Bacillus cereus*	CCM 2010	2789	2789	99%	0.0	99%
Strain no. 4	30%	*Bacillus tequilensis*	10b	2501	2501	96%	0.0	99%

### 3.2 Mosquito larvicidal activity

Probit analysis was carried out on the data obtained for the mosquito larvicidal activity, to calculate their LC_50_ values. As the mean to median ratio of the mortality data lies between 0.95 and 1.05, the data are assumed to be normally distributed. The probit method was used to calculate the LC_50_ using linear regression analysis on SPSS. The entire statistical analysis is given in Supporting Information 1. The LC_50_ values against *Culex quinquefasciatus* was 27.2 CFU/mL for *Bacillus paramycoides*, 34.2 CFU/mL for *Bacillus australimaris*, 39.1 CFU/mL for *Bacillus cereus* and 41.18 CFU/ml for *Bacillus tequilensis* as shown in Table 2. (CFU/mL – colony forming units per milliliter). If the bacterial mass of 1 bacterium is considered to be 1 pg,^28^, ^29^ then these values on conversion correspond to 27.2 × 10^‐3^ ng/mL for *Bacillus paramycoides*; 34.2 × 10^‐3^ ng/mL for *Bacillus australimaris*; 39.1 × 10^‐3^ ng/mL for *Bacillus cereus* and 41.18 × 10^‐3^ ng/mL for *Bacillus tequilensis*.

**TABLE 2:** Larvicidal toxicity of *Bacillus* species against the third instar stage of *Culex quinquefasciatus* mosquito larvae

		Larva (CFU/ml)	95% Fiducial Limit		
Bacillus species	% Toxicity	LC_50_	LFL	UFL	*Χ* ^2^ (*df *= 2)
*Bacillus paramycoides*	55%	27.2	18.23	40.3	3.62
*Bacillus australimaris*	40%	34.2	24.88	51.7	2.35
*Bacillus cereus*	35%	39.1	21.82	469.84	3.54
*Bacillus tequilensis*	30%	41.18	29.73	74.75	3.63

Abbreviations: LFL, lower fiducial limit; UFL, upper fiducial limit; CFU, colony‐forming units. Control, nil mortality.

### 3.3 SEM imaging for surface morphologies

**FIGURE 1:**
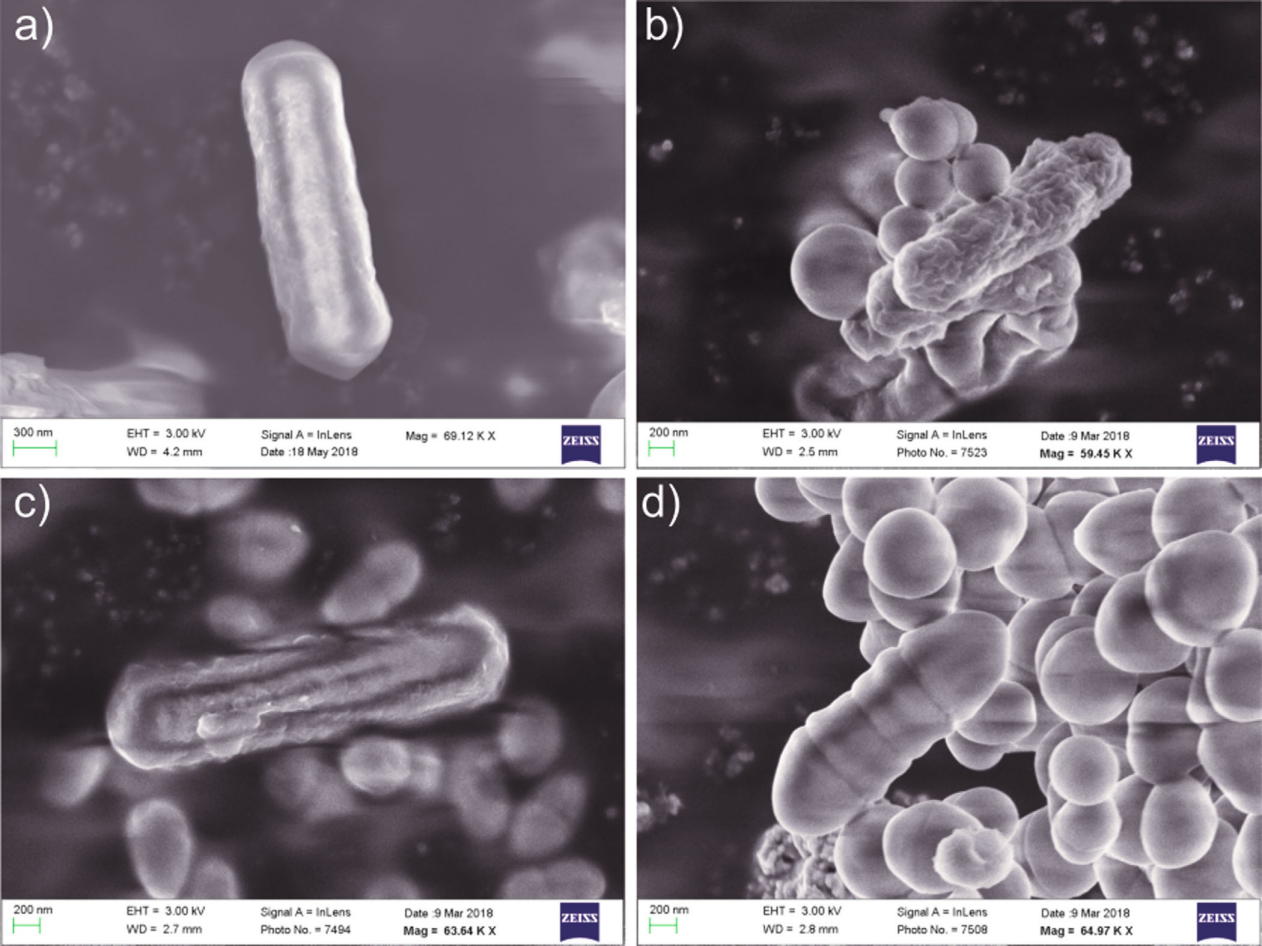
Scanning electron microscope images, captured at 3 kV, of the identified Bacillus species: A, *Bacillus paramycoides*; B, *Bacillus australimaris*; C, *Bacillus cereus*; and D, *Bacillus tequilensis*

To study the surface morphologies of the four identified *Bacillus sp*., they were imaged using Scanning electron microscopy. The SEM images showed *Bacillus paramycoides* to be cylindrically rod‐shaped with a width of 0.6 µm and a length of 1.5 µm (Figure 1A). As per the only literature available for the *B. paramycoides*, they too are rod‐shaped, belonging to the *B. cereus* group; ranging 0.8‐1.2 µm in width and 1.8‐2.2 µm in length.^30^ *Bacillus australimaris* showed roughness on its surface, with a width of around 0.6 µm and length of 1 µm (Figure 1B) that are similar to the morphological characteristics of *B. zhangzouensis* and *B. australimaris*.^31^ The SEM image of *Bacillus cereus* was found similar to that of *B. paramycoides* since both belong to the same group^30^ with a length of around 1.5 µm and width of 0.4 µm (Figure 1C). The uniqueness was found with the SEM images of *Bacillus tequilensis* that were tapering at both ends and showed variable segments on their surface (Figure 1D). To the best of our knowledge, such kind of morphology has never been reported to any of the reported *Bacillus* species images.

### 3.4 Proteins and MS data acquisition

The concentrations of the extracted proteins for *Bacillus paramycoides* was 6.16 µg/µL, *Bacillus australimaris* was 11.77 µg/µL*, B. cereus* was 12 µg/µL and *Bacillus tequilensis* was found to be 19 µg/µl. These samples were subjected to trypsin digestion prior to the LC‐MS/MS analysis.

The protein sequencing was carried out using LC‐MS. Peptide sequencing of *Bacillus paramycoides, Bacillus australimaris, Bacillus cereus*, and *Bacillus tequilensis's* proteome resulted in the acquisition of 14584 9236, 8553, and 7345 MS/MS spectra, respectively (Supporting Information 2‐5). These spectral peaks were compared with the UniProt protein databases of *Bacillus paramycoides* (Proteome ID: UP000182788)*, Bacillus australimaris* (Proteome ID: UP000050272)*, Bacillus cereus* (Proteome ID: UP000001417) and *Bacillus tequilensis* (Proteome ID: UP000250527) that resulted in the acquisition of no mosquitocidal toxins or any toxic proteins.

### 3.5 Proteins identified in customized database

As the search against the regular databases yielded no results, the high‐resolution MS/MS spectra were then searched against the customized database of the larvicidal toxin including Cry, Bin, VIP, and Mtx toxins.^32^

The acquired data of 14584 MS/MS spectra of *Bacillus paramycoides* when searched against the customized database of the toxins, we could identify 6 toxin protein groups (Supporting Information 2). The protein sequence coverage (PSC and Morpheus Scores (MS) for these peptides are shown in the parenthesis. These groups are Cry 41Ab (PSC: 7.72% and MS: 28.20), Cry 53Aa (PSC: 9.12% and MS: 28.08), VIP‐1 (PSC: 6.79% and MS: 21.12), Cry 57Aa (PSC: 7.88% and MS: 21.10), Cry 23Aa (PSC: 26.6% and MS: 21.10), and Bin B (PSC: 10.04% and MS: 12.07). The peptides identified in the protein sequences of *Bacillus paramycoides* mentioned above are presented in Supporting Information 2.

When the customized database was searched against the acquired data of 9236 MS/MS spectra of *Bacillus australimaris*, we can identify 11 toxin protein groups as shown in Supporting Information 3. These groups consists of Cry 57Aa (PSC: 11.82% and MS: 28.18), Cry 10Aa (PSC: 5.78% and MS: 23.12), Bin A (PSC: 20.54% and MS: 19.30), Cry 25Aa (PSC: 8.44% and MS: 16.22), Cry 64Aa (PSC: 12.46% and MS: 15.06), Cry 53Aa (PSC: 7.77% and MS: 13.09), Cry 26Aa (PSC: 2.49% and MS: 13.09), Cry 49Ab (PSC: 9.64% and MS: 12.25), Mtx2 (PSC: 13.01% and MS: 12.05), Cry 20Aa (PSC: 5.58% and MS: 12.03) and Cry 73 Aa (PSC: 6.34% and MS: 11.16). The peptides identified for *Bacillus australimaris* mentioned above are shown in Supporting Information 3.

Likewise, the search for the acquired data of 8553 MS/MS spectra of *Bacillus cereus* against the with the customized toxin database, led to the identification of six toxin protein groups (Supporting Information 4). They are Cry 4Ba, Cry4Ca, Cry4Aa (PSC: 5.81%, 3.9%, and 1.87% with MS: 26.20, 23.22 and 6.04 respectively), VIP–4 (PSC: 9.22% and MS: 37.22), Cry 22Aa, Cry 22Ba (PSC: 7.34%, 3.64% and MS: 17.13 and 10.23, respectively), Cry 63Aa (PSC: 10.62% and MS: 21.15), Cry 34Ab (PSC: 34.15% and MS: 21.14), and Cry 9Ca (PSC: 5.36% and MS: 19.14). The peptide sequences identified in the proteins of *Bacillus cereus* mentioned above are also highlighted in Supporting Information 4.

The 10 toxin groups were picked up when the acquired data of 7345 MS/MS spectra of *Bacillus tequilensis* was searched against the customized toxin database (Supporting Information 5). They are Mtx1 (PSC: 13.22%, MS: 37.36), Cry 50Ba, Cry 50Aa (PSC: 9.12% and 3.68% with MS: 16.08 and 10.05 respectively), Cry 43Ba (PSC: 6.98% and MS: 28.16), BinA (PSC: 21.89% and MS: 22.3), Cry 18Ca (PSC: 10.36% and MS: 19.07), Cry 16Aa (PSC: 5.71% and MS: 15.05), Cry 19Aa, Cry 19Ba (PSC: 7.6% and 2.79% with MS: 12.04 and 6.02 respectively), Cry 34 Ba, Cry 34Ab (PSC: 16.67% and 11.38% with MS: 11.06 and 7.02 respectively), Cry 60Aa, Cry 60Ba (PSC: 9.9% and 3.13% with MS: 11.04 and 6.03, respectively), and BinB (PSC: 7.37%, MS: 10.05). In case of *Bacillus tequilensis*, the identified peptide sequences are presented in Supporting Information 5. The summary of the identified peptide sequences of the larvicidal proteins, when searched against the customized toxin database, for all the four identified *Bacillus* species are shown in Table 3.

**TABLE 3:** The results of all mosquitocidal toxins obtained from LC‐MS after comparing it with the customized database that had all toxin template sequences of Cry (Cry1 to Cry73), Bin (BinA and BinB) VIP (VIP‐1, VIP‐2, VIP‐3, and VIP‐4), and Mtx (Mtx1, Mtx2, Mtx3)

	*Bacillus australimaris*		*Bacillus tequilensis*
*Bacillus paramycoides*	Bin A	*Bacillus cereus*	Bin A
Bin B			Bin B
		Cry 4Aa/ Ba	
		Cry 9 Ca	
	Cry 10 Aa		
			Cry 16 Aa
			Cry 18 Ca
			Cry 19 Aa/ Ba
	Cry 20 Aa		
		Cry 22Aa/ Ba	
Cry 23 Aa			
	Cry 25 Aa		
	Cry 26 Aa		
		Cry 34 Ab	Cry 34 Ab/ Ba
Cry 41 Ab			
			Cry 43 Ba
	Cry 49 Ab		
			Cry 50 Aa/Ba
Cry 53 Aa	Cry 53 Aa		
Cry 57 Aa	Cry 57 Aa		
			Cry 60 Aa/ Ba
		Cry 63 Aa	
	Cry 64 Aa		
	Cry 73 Aa		
			Mtx 1
	Mtx 2		
VIP‐1			
		VIP‐2	

### 3.6 In silico analysis

In‐silico analysis of all toxin template sequences of Cry (Cry1 to Cry73), Bin (BinA and BinB) VIP (VIP1, VIP2, VIP3, and VIP4), and Mtx (Mtx1, Mtx2, Mtx3) were independently searched against the database of *Bacillus paramycoides*, *Bacillus australimaris, Bacillus cereus*, and *Bacillus tequilensis* using p‐BLAST with default parameters. The toxin sequences with a large number of hits and significant E‐values (E‐values ≤ −10) were considered. The following Table 4 shows the final results for the presence of respective toxin template sequences in the four *Bacillus sp*. According to the NCBI protein database, *Bacillus paramycoides* exhibit Cry 5, Cry 8, and Cry 16 isoforms of Cry toxins, Mtx‐3, and VIP‐1 and VIP‐4. *Bacillus australimaris* exhibits only Cry 32 toxin but completely lacks the VIP, Mtx, and Bin toxins. The *Bacillus tequilensis* database showed the presence of BinA, VIP‐2 and VIP‐3. On the other hand, *Bacillus cereus* showed hits with all the known toxins except Cry 34, and Cry 38 that had substantial E‐scores are shown in Table 4.

**TABLE 4:** The sequences of the known toxins identified using p‐BLAST search in the identified *Bacillus* species using template sequences of Cry (Cry1 to Cry73), Bin (BinA and BinB) VIP (VIP‐1, VIP‐2, VIP‐3, and VIP‐4), and Mtx (Mtx1, Mtx2, Mtx3)

		*Bacillus cereus*	
		Bin A	Bin A
*Bacillus paramycoides*	*Bacillus australimaris*	Bin B	*Bacillus tequilensis*
Cry5Ca			
Cry 8Ea			
		No Cry 34 & Cry 38 Rest all Cry are present	
Cry16Aa			
	Cry32Da		
		Mtx‐1	
Mtx‐3		Mtx‐2	
		Mtx‐3	
VIP‐1		VIP‐1	
		VIP‐2	VIP‐2
VIP‐4		VIP‐3	VIP‐3
		VIP‐4	

After comparing the search results of the toxins against the customized database with that of the in‐silico p‐BLAST database, no common toxins were found in the case of *Bacillus australimaris* data. The presence of VIP‐1 in *Bacillus paramycoides*, BinA in *Bacillus tequilensis* and Cry 4, Cry 9, Cry 22, Cry 63, and VIP‐2 in *B. cereus* were obtained in both the results thereby confirming their presence in their host.

### 3.7 Metabolites and MS data acquisition

The metabolites extracted from the *Bacillus sp*. were subjected to LCMS for their analysis which resulted in the acquisition of nearly 327, 288, 366, and 209 metabolites from *Bacillus cereus*, *Bacillus paramycoides, Bacillus tequilensis*, and *Bacillus australimaris* respectively (Supporting Information 6). The metabolites mentioned were obtained following the search against the available database with a high cutoff value of 99.5% was selected and are reported. Also, these metabolites were individually analyzed for their role in distinct biochemical pathways using the online tool KEGG (https://www.genome.jp/kegg/pathway.html). There were a substantial number of metabolites that were found common amongst the four *Bacillus* species. The metabolites which are found common in at least three of the *Bacillus* species are Terephthalic acid; 1‐octadecanoyl‐rac‐glycerol; Betaine; 3,7‐EPOXYCARYOPHYLLAN‐6‐ONE; 2‐methyl lauric acid; 11‐hydroxy palmitic acid; C8‐Dihydroceramide; and 1alpha,25‐dihydroxy‐2beta‐ (6‐hydroxyhexyl)vitamin D3/1alpha,25‐dihydroxy‐2beta‐ (6‐hydroxyhexyl)cho. These metabolites are highlighted in yellow in Supporting Information 6. Apart from the aforementioned metabolites, there are many small compounds identified that are common in at least two, highlighted in cyan in Supporting Information 6, of the *Bacillus* bacteria. For the rest of the unique metabolites identified a majority of them belonged to the class of the unsaturated fatty acids, amides, or complex alcohols.

## 4 DISCUSSION

The major aim of this study was to identify bacteria from the wild having larvicidal activity and can be potentially used as an alternative well‐known *Bacillus thuringiensis* and *Bacillus sphaericus*, which could be effectively used further for vector control. We carried out the toxicity assays against *Culex quinquefasciatus* (third instar) using our isolated gram‐positive bacterium and we observed four Bacillus isolates (*Bacillus paramycoides, Bacillus australimaris, Bacillus cereus*, and *Bacillus tequilensis*) to exhibit larvicidal activity. These Bacillus species were unknown and were found to be unique in terms of mosquito larvicidal activity. The respective larvicidal activity values repoted in terms of LC_50_ values against *C. quinquefasciatus* were 27.2 CFU/mL for *Bacillus paramycoides*, 34.2 CFU/mL for *Bacillus australimaris*, 39.1 CFU/mL for *Bacillus cereus*, and 41.18 CFU/mL for *Bacillus tequilensis* (Table 2). These LC_50_ values on conversion correspond to 27.2 × 10^−3^ ng/mL for *Bacillus paramycoides*; 34.2 × 10^−3^ ng/mL for *Bacillus australimaris*, 39.1 × 10^−3^ ng/mL for *Bacillus cereus*, and 41.18 × 10^−3^ ng/mL for *Bacillus tequilensis*, if a bacterial mass of 1 CFU is considered to be 1 pg. It should be noted that standard toxicity, available in the literature, against *Culex* mosquito larvae for *Bacillus thuringiensis* ranges from 0.5 to 1.47 ng/mL and that for *Bacillus sphaericus* is around 3.0 ng/mL.^28^, ^29^ Our LC_50_ data indicate that our *Bacillus* strains are almost 100 times more potent in terms of larvicidal activity against *culex*, when compared with the well‐established *Bti*.^33^ Our findings allowed us to report that even our minimum toxic strain (30%) of *Bacillus tequilensis* is almost 10‐folds more toxic than the standard *Bacillus thuringiensis* and *Bacillus sphaericus* strains. We also report, for the very first time, that *B. paramycoides, B. australimaris, B. cereus*, and *B. tequilensis*, exhibits substantial larvicidal toxicity against *Culex* mosquito. All the tests and controls were performed in duplicates and the readings are the average of three independent experiments. Two sets of control, one with mosquito larvae feed and another without the feed were used. Control with larvae feed was kept to avoid standard error in mortality due to starvation. Only those experiments were considered wherever both these controls showed no mortality. This means that we had done many experiments than what we had reported in the manuscript to ensure that only those sets are used, where both of our controls are perfect.

Through in silico analysis, we could trace back the presence of the established Cry, Mtx, Bin, and VIP mosquitocidal proteins in the respective *Bacillus sp*. and then verified it after comparing them with our practical proteomic data through LC‐MS.

To investigate the active components responsible for these toxicities, we undertook the proteomic approach by profiling the full cell extract of these bacteria using LC‐MS. The LC‐MS of *Bacillus paramycoides, Bacillus australimaris, Bacillus cereus*, and *Bacillus tequilensis* proteome resulted in the acquisition of 14584 9236, 8553, and 7345 MS/MS spectra, respectively. The acquired data were further analyzed using Morpheus and led to no match corresponding to any of the established mosquito larvicidal toxins when we search our spectral peaks against the protein database for the respective *Bacillus sp*. linked to the software. Further, we customized our database comprising of all the established *Bacillus* toxins (Cry, Mtx, VIP, and Bin) and searched our MS/MS spectra for our bacterium using default parameters in Morpheus. In our mass‐spec data analysis, we could identify six peptides corresponding to four isoforms of Cry toxins and 1 each of VIP‐1 and BinB toxin in *Bacillus paramycoides*. For *Bacillus australimaris* 11 peptides showed high similarity to nine isoforms of Cry toxins and one each of BinA and Mtx2 toxin. In case of *B. cereus*, six peptides belonged to five isoforms of Cry toxins and one VIP‐2 toxin. Finally, 10 peptides belonging to seven isoforms of Cry toxins, one belonging to Mtx1 and two belonging to the functional BinA‐B toxin in the strain *Bacillus tequilensis*. These toxic components could only be picked up when analyzed against our customized database with high confidence.

It is quite evident from Table 3, that the toxicity for *Bacillus paramycoides, Bacillus australimaris*, and *B. cereus* is not likely because of the Binary toxin system of BinA and BinBbecause of the highly acceptable fact that the binary toxins BinA‐BinB of *Lysinibacillus sphaericus* shows mosquito larvicidal activity as a heterodimer in a stoichiometric mixture of 1:1.^34^ Through this information one can conveniently infer that the two components are active only when both are expressed in equal proporitions and hence both BinA and BinB should have been detected in our mass spectra. Our data analysis reveals that no Binary toxins are present in *B. cereus* and only one of the components is expressed in our other two bacterial species. For *Bacillus paramycoides* we could identify only BinB, the inactive component of the complex. However, for *Bacillus australimaris* we got the toxic component BinA, whose activity is known to be highly reduced in the absence of its interacting partner, BinB.^34^ The presence of both BinA and BinB in *Bacillus tequilensis* might be the plausible reason for its 30% toxicity against *C. quinquefasciatus* mosquito larvae that needs further validation.

For *Bacillus paramycoides* we also identified the presence of VIP‐1 and VIP‐2 for *B. cereus*. Similar to Binary toxins, VIP‐1 and VIP‐2 have also been reported to exhibit high toxicities against some pests. Their significant homology with the binary toxins suggests that VIP‐1 and VIP‐2 might also form typical A+B type binary toxins, where VIP‐2 is the cytotoxic A‐domain and VIP‐1 the receptor‐binding domain responsible for the translocation of the cytotoxic VIP‐2 into the host cell.^35^ This system needs further investigation and commenting on the stoichiometric aspect of this heterodimer is beyond the scope of this manuscript.

Cry 23Aa found in *Bacillus paramycoides* is a small protein composed of β‐strands that associate with the protein cry 37 and form a binary pore‐forming toxin active against some Coleopteran insects.^36^ There are additional Cry family proteins like Cry31A, Cry41A, Cry45A, Cry46A, Cry63A, and Cry64A, which has been reported to exhibit significant and specific cytocidal activity against human cancer cells of various origins. These proteins have been given the alternative names parasporin‐1 (PS1), parasporin‐3 (PS3), parasporin‐4 (PS4), parasporin‐2 (PS2), parasporin‐6 (PS6), and parasporin‐5 (PS5), respectively.^35^ Recently, a study revealed that a novel amino acid Cry‐related protein which share ∼40% sequence identity with cancer cell killing Cry proteins parasporins Cry41Ab1 and Cry41Aa1, also has some insecticidal activity.^37^ Interestingly we have also recognized Cry 41Ab1 in *Bacillus paramycoides* as well and this makes it difficult to pinpoint the real component responsible for exhibiting the larvicidal activity amongst Cry 23Aa; Cry 41 Ab1; and VIP‐1.

Similarly, the presence of Cry 63 (PS6) and Cry 64 (PS5) in *Bacillus cereus* and *Bacillus australimaris* respectively, might be the reason for their medieval toxicity against *C. quinquefasciatus*.

In our study, *B. cereus* has also shown the presence of both Cry 4 and Cry 9 toxins along with VIP‐2. There have been reports that indicate that Cry4 toxin protein bound on the mineral crystalline retained its larvicidal activity against *Aedes aegypti*.^38^ Also, there has been a report which states that, when applied together, the three‐domain Cry toxin, Cry9Aa, and the VIP, VIP3Aa, exhibited high insecticidal activity against an insect pest, the Asiatic rice borer (*Chilo suppressalis*).^39^ They also found that the Cry 9 and VIP toxins have unique binding sites specifically to brush border membrane vesicles of *C. suppressalis* whose synergistic activities are potent and have effective insect control which also delays the development of insect resistance. The presence of the Cry 4 and Cry 9toxins along with VIP‐2 in *B. cereus* could be the main reason for their toxicity.

For the commonly expressed proteins in the two‐bacterial systems *Bacillus paramycoides* and *Bacillus australimaris*, Cry53Aa and Cry57Aa, we do not have any literature available that can link them to mosquito larvicidal activity. Whereas the structural studies of Cry 34Ab1 that is commonly expressed for *B. cereus* and *Bacillus tequilensis* reveal that Cry 35Ab1 binds to the Western Corn Rootworm brush border membrane vesicles that are dramatically enhanced by the presence of Cry 34Ab1 for its pore‐forming characteristic.^40^, ^41^ Also, the very fact that all these bacteria are showing different toxicity levels could indicate that these Cry proteins may not be directly responsible for any kind of toxicity. However, one cannot rule out the possibility of the role of these proteins as regulators in toxicity cascade, which needs further validation.

For the other candidates of the Cry family, that is, Cry 10 Aa, Cry 16Aa, Cry 18Ca, Cry 20 Aa, Cry 22Aa, Cry 25 Aa, Cry 26 Aa, Cry 43Ba, Cry 49 Ab, Cry 50Ab/Ba, Cry 60 Ab/Ba, and Cry 73 Aa we could not find any concrete literature (s) which could link these identified proteins to larvicidal activity.

The presence of mosquitocidal toxin, Mtx‐2 in *Bacillus australimaris* and Mtx‐1 in *Bacillus tequilensis* is quite interesting as Mtx proteins are produced during vegetative growth and are known to enhance the activity of other toxins and to reduce the expression of insecticide resistance.^42^

In addition to individual protein function, we also carried out metabolite analysis of the bacteria using LC‐MS. The results of the differential metabolomics obtained were analyzed by KEGG pathways in *Bacillus sp*. Analysis of KEGG pathway revealed that the acquired metabolites above the 99.5% confidence limit were majorly involved in metabolic pathways, biosynthesis of secondary metabolites, and microbial metabolism in diverse environments, carbon metabolism, and citrate cycle. After comparing all the metabolites, a major fraction of the metabolites was found to be present in all the *Bacillus* species and are thus very unlikely to be responsible for any have any role in the mosquitocidal toxicity. Further, the other unique metabolites, they belonged to the class of either unsaturated fatty acids, amides, or complex alcohols and thus seem to be unlikely candidates playing any role in larvicidal activity. Also, it was found that none of these metabolites were related to any kind of toxins, that is, none of the Cry, Mtx, binary, or VIP proteins were found to be regulated in any of pathways involving these metabolites. None of the metabolites showed any significance in terms of any kind of toxicity apart from their basic cellular biochemical pathways. This led us to comment that the differential toxicity in them might be due to their differential proteome and not their metabolome. However, we do not completely rule out the possible role of the metabolites in the mosquito larvicidal activity because of the lack of literature available on these lines and hence requires further investigation.

## 5 CONCLUSIONS

The four bacterial strains reported in this study were isolated from the mid‐gut of dead *C. quinquefasciatus* mosquito larvae collected from the various wild location of the city of Mumbai (India). They were tested for their larvicidal activity against the laboratory cultured, third instar *Culex* mosquito larvae. These toxicities varied from a minimum of 30% to 55%. These bacteria were identified by 16S rRNA sequencing technique and all four bacteria were found to be distinct strains of *Bacillus* species. The surface morphologies of these identified bacteria were imaged by Scanning Electron Microscope for their (Bacilli) cylindrical morphology, which further supports our sequencing results. Our toxicity assay findings allowed us to report that even our minimum toxic strain (30%) of *Bacillus tequilensis* is almost 10‐fold more toxic than the usual *Bacillus thuringiensis* and *Bacillus sphaericus* strains.

To the best of our knowledge, we are the first to report the proteomic profiles of *Bacillus paramycoides, Bacillus australimaris, Bacillus cereus*, and *Bacillus tequilensis* isolated from the mid‐gut of *Culex*. The present study attempted in identifying *Bacillus paramycoides, Bacillus cereus, Bacillus australimaris*, and *Bacillus tequilensis* to be potent mosquitocidal strains. To the best of our knowledge none of these strains have reported earlier for having larvicidal toxicity. At the proteomics level, we tried to identify various peptide sequences, which corresponded to respective toxin proteins in these *Bacillus* species from our data. Interestingly, we could identify that either of the Cry 23Aa, Cry 41 Ab1, and VIP‐1 in *Bacillus paramycoides*; Cry 64Aa (PS5) or Mtx‐2 in *Bacillus australimaris*; Cry 4Aa/Ba, Cry 9Ca along with VIP‐2 in *B. cereus* and BinA‐BinB along with Mtx‐1 proteins in *Bacillus tequilensis* are toxic by some or the other function in nature in different systems and therefore might be the reason for their toxicity. The in silico approach was used to further confirm the presence of the known toxins in them that revealed that only VIP‐1 in *Bacillus paramycoides*, BinA in *Bacillus tequilensis* and Cry 4, Cry 9, Cry 22, Cry 63, and VIP‐2 in *B. cereus* were obtained in both the in‐silico as well as in their LC‐MS results thereby confirming their presence in their host. Non‐identification of other toxins from the p‐BLAST database in our LC‐MS analysis could be because of the smaller intensity of the spectral peaks owing to low expression levels of these toxins in Gram‐positive bacterium compared to the highly expressing housekeeping proteins. Or it might also be possible to say that the NCBI protein database needs an upgradation for *Bacillus australimaris* and *Bacillus tequilensis* as our LC‐MS data clearly show the presence of the mosquitocidal toxins. Interestingly, one should consider *Bacillus cereus* as a potential gram‐positive bacterium to act, in general as insecticidal species. Both our LC‐MS data and bioinformatics analysis of *Bacillus cereus* confirm the presence of almost Cry toxins that have been majorly reported for *Bacillus thuringenesis* and also of binary (Bin) toxins mainly known to have been present in *Bacillus sphericus*. This raises a question about why *Bacillus cereus* till date has not been popular with entomologists to be used as a super‐insecticidal bacterium. Owing to the low mosquito‐cidal toxicity of *Bacillus cereus*, it would be interesting to find out the mechanistic pathway for the same, which might be keeping Cry; Mtx; VIPs and/or Bin in dormant form and prevents them to act against the mosquito larvae. However, work needs to be done to test their efficiency and efficacy against the other agricultural pests and insects.

Finally, we suggest that our findings serve as a baseline and open new avenues for further research in the field of mosquito larvicidal toxins. It would be interesting to further understand both increasing the activity of commercial bacterial larvicides and managing potential resistance to these substances among mosquito populations. Also, the other less studied Cry proteins like Cry 10 Aa, Cry 16Aa, Cry 18Ca, Cry 20 Aa, Cry 22Aa, Cry 25 Aa, Cry 26 Aa, Cry 43Ba, Cry 49 Ab, Cry 50Ab/Ba, Cry 60 Ab/Ba, and Cry 73 Aa needs more attention from the research community to better understand the entire regulatory cascade responsible for the larvae/insecticidal toxicity exhibited by these gram‐positive bacteria.

## AUTHOR CONTRIBUTION

Domnic Colvin designed, executed experiments, analyzed the data, and written the manuscript. Mahesh Samant performed the statistical analysis and database preparation. H. N. Verma and Rama Lokhande jointly co‐supervised this work. Avinash Kale designed, analyzed the data, and written the manuscript

## CONFLICT OF INTEREST

The authors declare that there is no conflict of interest.

## Supporting information

Supporting Information

Supporting Information

Supporting Information

Supporting Information

Supporting Information

Supporting Information
