# Supplementary material for: Proteomic and metabolomic approach to rationalize the differential mosquito larvicidal toxicity in Bacillus sp. isolated from the mid‐gut of Culex quinquefasciatus mosquito larvae
Source: Anal Sci Adv. 2020 Oct 12;2(11-12):505–14. doi: 10.1002/ansa.202000081 (PMC10989537; doi:10.1002/ansa.202000081)
Supplement: Supplementary file 1 — Supporting Information [file ANSA-2-505-s003.docx]

| **Site** | **Latitude** | **Longitude** | **Strains** | **Identified Species** |
| --- | --- | --- | --- | --- |
| 1 | 19°N 23ʹ 31.794ʺ | 72°E 49ʹ 13.871ʺ | Strain no.3 & Strain no. 4 | *B. cereus* & *B. tequilensis* |
| 2 | 19°N 18ʹ 44.293ʺ | 72°E 50ʹ 59.868ʺ | Strain no. 1 & Strain no. 2 | *B. paramycoides* &  *B. australimaris* |

**Supplementary data 1:** The location co-ordinates for the collection sites from where the dead Culex mosquito larvae were collected.
