# Supplementary material for: Proteomic and metabolomic approach to rationalize the differential mosquito larvicidal toxicity in Bacillus sp. isolated from the mid‐gut of Culex quinquefasciatus mosquito larvae
Source: Anal Sci Adv. 2020 Oct 12;2(11-12):505–14. doi: 10.1002/ansa.202000081 (PMC10989537; doi:10.1002/ansa.202000081)
Supplement: Supplementary file 2 — Supporting Information [file ANSA-2-505-s004.docx]

| **Sr. No.** | **Protein Id** | **Protein Name** | **Number of Peptide-Spectrum Matches** | **Summed Unique Peptide Precursor Intensity** | **Protein Sequence Coverage (%)** | **Summed Morpheus Score** |
| --- | --- | --- | --- | --- | --- | --- |
| 1 | Q6BE06 | cry 41 Ab | 4 | 31767.70 | 7.72 | 28.20 |
| 2 | E6Y2M1 | cry 53 Aa | 4 | 30701.23 | 9.12 | 28.08 |
| 3 | V9HXE2 | Vip 1 | 3 | 25375.00 | 6.79 | 21.12 |
| 4 | C0LUW0 | cry 57 Aa | 3 | 16507.07 | 7.88 | 21.10 |
| 5 | Q9KKG8 | cry 23 Aa | 3 | 25601.90 | 26.60 | 21.10 |
| 6 | B0LUQ9 | Bin B | 2 | 26371.80 | 10.04 | 12.07 |

**Supplementary Data 2**: Results of the toxin proteins identified in customized database from 14,584 MS/MS spectra of *Bacillus paramycoides*

The unique peptides identified in the proteins of *Bacillus paramycoides* corresponding to larvicidal toxins are highlighted in the sequences

1. **cry41Ab** gi|51090236|dbj|BAD35163.1| cancer cell-killing Cry protein [Bacillus thuringiensis]

MNQSCNNNGYEVLNSGKGYCQPRYPFAQAPGSELQNMGYKEWMNMCTSGDPTVLGGGYSADVKDAVITSI NIASYLLSVPFPPAGVAAGILGALLGLLWPTNTQAVWEAFMNTVEALINQKLDEYARSKAISELNGLKNV LELYQDAADDWNENPGDLRNKNRVLTEFRNVNGHFENSMPSFAVRNFEVNLLPVYAEAANLHLLLLRDAV KFGEGWGMSTDPGAERDDMYRRLRSRTEIYTDHCVNTYNQGLQQAKSLQANVSDYSRYPWTQYNQSGGFS YREAKGEYRGTENWNLYNAFRRDMTILVLDIIAQFPTYDPGLYSRPVKSELTREVYTDIRGTTWRSDANL NTIDAIENRMVGSRQLQLFTWLTEMKFYIRNTGSITSYTHGDLMVGLEKKIRKTNDNDQWLPLEGQNTSY TRIDRPGIELGKNYWYYARTQQWFETRLLQLWVNTDVLSLNAGTVGNEFWARDVPDYRNIYARSTRNHFI ENHRLSWIKFEPVRDNCPFAWPGYKQLSALLFGWTHNSVDPFNTIASDRITQIPAVKGYLVDNGATVVRG PGNTGGDLVRLPAYNQQWTQLRVKVRPSTTARTRGYNVRIRYASEGNANLFVGKYVDTANRFYETGNYAV NQTFSGSMTYNSFKYLDAIGFAANEEEFRIELRCNSGGPIYIDKIEFIPVNPIPEPPEGIYQIVTALNNS SVVTSEEFCMGIGLTTRCGVNLWSNNGNTLQKWRFVYNGDQNAFQIKSTPNEDLVLSGSNSGTSVTAETN QNRPNQHWLIEEAGNGYVYLRSKGNPNLVLDVAGTSTANGTNIILWNYNGSTNQKFKLS

1. **Cry53Aa** gi|157418804|gb|ABV55105.1| Cry toxin [Bacillus thuringiensis]

MNSYQNKNEYEILDASQNNSTMSNHYPRYPLAKDPLASMQNTNYKDWLNLCDTPNMENPEFQSVGRSALSILINLSSKILSLLGIPFAAQIGQLWSYTLNLLWPVANNATQWDIFMRTIEDLINVRIETSVRNRALAELEGLGNILEDYKVALRRWDLNPTNLDRQSEVVSQFEIVHAFFRVQMPVFAIRDFEVPLLPVYASAANLHLLLLRDVVINGDRWGLSAARINDYHDLQLRLTSTYTDHCVNWYNTGLNRLIGTNARQWVTYNQFRREMTISVLDIISLFSNYDARRYPTKTQSELTRMIYTDPIGAVGTIGLNPGWLDNAPSFSVIENSVVQSPRTFLFLERVGIFTGVLHGWSSQSQFWSAHRLFLSNLSSIWESIIYGNPQNNIGYEEVDFTNFDVFSINSRATSIMSPFGGGELFGVPRVTFDLSNRTNNSLAQRTYNRPFTFGGQDIVSRLPGETTEIPNSSNFSHRLAYISSFRVGIAGSVLSYGWTHHSVDRHMRLNPNMITQIPAVKXVSGHIVSGPGHTGGDILRVHSGSQGTIIIQSNSAQRYRLRLRYSSTLPGDLILNHRGTDGSQQFIEFTLPATSGQLRFADFTYADGRTVFQTPNSHVFYTVHVQTRSNGIFFIDKIDYIPENTPPLECGGERNLEKEKKAVNDLFTN

1. >ADK79125.1 vegetative insecticidal protein 2 [Bacillus thuringiensis]

MKNMKKKLASVVTCTLLAPMFLNGNVNAVYADSKTNQISTTQKNQQKEMDRKGLLGYYFKGKDFSNLTMFAPTRDST LIYDQQTANKLLDKKQQEYQSIRWIGLIQSKETGDFTFNLSEDEQAIIEINGKIISNKGKEKQVVHLEKGKLVPIKI EYQSDTKFNIDSKTFKELKLFKIDSQNQPQQVNQDELRNPEFNKKESQEFLAKPSKINLFTQKMKREIDEDTDTDGD SIPDLWEENGYTIQNRIAVKWDDSLASKGYTKFVSNPLESHTVGDPYTDYEKAARDLDLSNAKETFNPLVAAFPSVN VSMEKVILSPNENLSNSVESHSSTNWSYTNTEGASVEAGIGPKGISFGVSVNYQHSETVAQEWGTSTGNTSQFNTAS AGYLNANVRYNNVGTGAIYDVKPTTSFVLNNDTIATITAKSNSTALNISPGESYPKKGQNGIAITSMDDFNSHPITL NKKQVDNLLNNKPMMLETNQTDGVYKIKDTHGNIVTGGEWNGVIQQIKAKTASIIVDDGERVAEKRVAAKDYENPED KTPSLTLKDALKLSYPDEIKEIEGLLYYKNKPIYESSVMTYLDENTAKEVTKQLNDTTGKFKDVSHLYDVKLTPKMN VTIKLSILYDNAESNDNSIGKWTNTNIVSGGNNGKKQYSSNNPDANLTLNTDAQEKLNKNRDYYISLYMKSEKNTQC EITIDGEIYPITTKTVNVNKDNYKRLDIIAHNIKSNPISSIHIKTNDEITLFWDDISITDVASIKPENLTDSEIKQI YSRYGIKLEDGILIDKKGGIHYGEFINEASFNIEPLQNYVTKYKVTYSSELGQNVSDTLESDKIYKDGTIKFDFTKY SKNEQGLFYDSGLNWDFKINAITYDGKEMNVFHRYNK

1. **cry57Aa** gi|225348555|gb|ACN87261.1| Cry delta-endotoxin [Bacillus thuringiensisserovarkim]

MGTWWPTDSASDTWGEMIGFAQELVGTALSEDLKIRTNQQIDSIRIALQAYYSSLEDWLNANKPLSGPLLNQVTEEFGNALRKSRDSIAYFKSDDSNVYTIILLPAYAQVANFHLALIHEGLKYATEWNLPRLQTFGYEEDLKHYTISYVNHCEYWYQKGLDILYPRNVIGMTQWMKRNFYRLNMTINVLDIISLFSLYDSKKYPNFFEDIYNAQKELISKFQLTRIVTTEPTLHQHKYLNDSSKKICQNESSCDPIDLDEYLTLPLMFQNWLRNINFQYLAPVISVGDEALYPFFVATQNINEYMNAEGNMIIGRQQGLWNFQFIIVHLFHLVYKKMTIFMVKWLSAYPLIDEDILNGVRTPYILQKIEFYNLNKSINSKQIRPISAGTTKSPLIDIYYGLPDVNGYNLDEPQFNFNAASHYFNSIQTCYYKENTSKNHYDIYQSYVFHWEHASVKRKDEVVSDRITIFPAIKSNPILSRGIQIISHQGHTGGNVIYFTPQSELHFKINFVSNRQKYKIRLRYVAFNPVVIQYHGSNSYASLSSITLPRTSSNQNVRDLRYEEFGYSDFEINMSSAGGLEDIKIISNNEFILDRIEFIPDTLFNYLS

1. **cry23Aa** gi|8567977|gb|AAF76375.1|AF038048_1 crystal protein [Bacillus thuringiensis]

MGIINIQDEINNYMKEVYGATTVKSTYDPSFKVFNESVTPQFTEIPTEPVNNQLTTKRVDNTGSYPVESTVSFTWTETHTETSAVTEGVKAGTSISTKQSFKFGFVNSDVTLTVSAEYNYSTTNTTTTTETHTWSDSTKVTIPPKTYVEAAYIIQNGTYNVPVNVECDMSGTLFCRGYRDGALIAAVYVSVADLADYNPNLNLTNKGDGIAHFKGSGFIEGAQGLRSIIQVTEYPLDDNKGRSTPITYLINGSLAPNVTLKNSNIKF

1. **BinB** gi|166034391|gb|ABY78896.1| **binary toxin B [Lysinibacillussphaericus]**

MCDSKDNSGVSEKCGKKFTNYPLNTTPTSLNYNLPEISKKFYNLKNKYSRNGYGLSKTEFPSSIENCPSNEYSIMYDNKDPRFLIRFLLDDGRYIIADRDDGEVFDEAHTYLDNNNHPIISRHYTGEERQKFEQVGSGDYITGEQFFQFYTQNKTRVLSNCRALDSRTILLSTAKIFPIYPPASETQLTAFVNSSFYAAAIPQLPQTSLLENIPEPTSLDDSGVLPKDAVRAVKGSALLPCIIVHDPNLNNSDKMKFNTYYLLEYKEYWHQLWPQIIPAHQTVKIQERTGISEVVQNSMIEDLNMYIGADFGMLFYFRSSGFKEQITRGLNRPLSQTTTQLGERVEEMEYYNSNDLDVRYVKYALAREFTLKRVNGEIVKNWVAVDYRLAGIQSYPNAPITNPLTLTKHTIIRCENSYDGHIFKTPLIFKNGEVIVKTNEELIPKINQ
