# Supplementary material for: Proteomic and metabolomic approach to rationalize the differential mosquito larvicidal toxicity in Bacillus sp. isolated from the mid‐gut of Culex quinquefasciatus mosquito larvae
Source: Anal Sci Adv. 2020 Oct 12;2(11-12):505–14. doi: 10.1002/ansa.202000081 (PMC10989537; doi:10.1002/ansa.202000081)
Supplement: Supplementary file 3 — Supporting Information [file ANSA-2-505-s006.docx]

| **Sr. No.** | **Protein Id** | **Protein Name** | **Number of Peptide-Spectrum Matches** | **Summed Unique Peptide Precursor Intensity** | **Protein Sequence Coverage (%)** | **Summed Morpheus Score** |
| --- | --- | --- | --- | --- | --- | --- |
| 1 | C0LUW0 | cry 57 Aa | 6 | 10648.20 | 11.82 | 28.18 |
| 2 | P09662 | cry 10 Aa | 4 | 10055.46 | 5.78 | 23.12 |
| 3 | Q0MQM7 | Bin A | 5 | 8488.39 | 20.54 | 19.30 |
| 4 | O87906 | cry 25 Aa | 3 | 9078.42 | 8.44 | 16.22 |
| 5 | D4QGQ7 | cry 64 Aa | 3 | 14707.65 | 12.46 | 15.06 |
| 6 | E6Y2M1 | cry 53 Aa | 2 | 5329.14 | 7.77 | 13.09 |
| 7 | Q9X597 | cry 26 Aa | 2 | 3145.70 | 2.49 | 13.09 |
| 8 | A7WKY9 | cry 49 Ab | 2 | 5695.15 | 9.64 | 12.25 |
| 9 | Q45470 | mtx 2 | 2 | 11816.86 | 13.01 | 12.05 |
| 10 | O32321 | cry 20 Aa | 2 | 52959.52 | 5.58 | 12.03 |
| 11 | M1E9P2 | cry 73 Aa | 2 | 6677.27 | 6.34 | 11.16 |

**Supplementary data 2**: Results of the toxin proteins identified in customized database from 9,236 MS/MS spectra of *Bacillus australimaris*

The unique peptides identified in the proteins of *Bacillus australimaris* corresponding to larvicidal toxins are highlighted in the sequences

1. >**cry57Aa** gi|225348555|gb|ACN87261.1| Cry delta-endotoxin [Bacillus thuringiensis serovar kim]

MGTWWPTDSASDTWGEMIGFAQELVGTALSEDLKIRTNQQIDSIRIALQAYYSSLEDWLNANKPLSGPLLNQVTEEFGNALRKSRDSIAYFKSDDSNVYTIILLPAYAQVANFHLALIHEGLKYATEWNLPRLQTFGYEEDLKHYTISYVNHCEYWYQKGLDILYPRNVIGMTQWMKRNFYRLNMTINVLDIISLFSLYDSKKYPNFFEDIYNAQKELISKFQLTRIVTTEPTLHQHKYLNDSSKKICQNESSCDPIDLDEYLTLPLMFQNWLRNINFQYLAPVISVGDEALYPFFVATQNINEYMNAEGNMIIGRQQGLWNFQFIIVHLFHLVYKKMTIFMVKWLSAYPLIDEDILNGVRTPYILQKIEFYNLNKSINSKQIRPISAGTTKSPLIDIYYGLPDVNGYNLDEPQFNFNAASHYFNSIQTCYYKENTSKNHYDIYQSYVFHWEHASVKRKDEVVSDRITIFPAIKSNPILSRGIQIISHQGHTGGNVIYFTPQSELHFKINFVSNRQKYKIRLRYVAFNPVVIQYHGSNSYASLSSITLPRTSSNQNVRDL RYEEFGYSDFEINMSSAGGLEDIKIISNNEFILDRIEFIPDTLFNYLSN

1. >**cry10Aa** gi|143229|gb|AAA22614.1| insecticidal endotoxin (put.); putative [Bacillus thuringiensis]

MNPYQNKNEYEIFNAPSNGFSKSNNYSRYPLANKPNQPLKNTNYKDWLNVCQDNQQYGNNAGNFASSETIVGVSAGIIVVGTMLGAFAAPVLAAGIISFGTLLPIFWQGSDPANVWQDLLNIGGRPIQEIDKNIINVLTSIVTPIKNQLDKYQEFFDKWEPARTHANAKAVHDLFTTLEPIIDKDLDMLKNNASYRIPTLPAYAQIATWHLNLLKHAATYYNIWLQNQGINPSTFNSSNYYQGYLKRKIQEYTDYCIQTYNAGLTMIRTNTNATWNMYNTYRLEMTLTVLDLIAIFPNYDPEKYPIGVKSELIREVYTNVNSDTFRTITELENGLTRNPTLFTWINQGRFYTRNSRDILDPYDIFSFTGNQMAFTHTNDDRNIIWGAVHGNIISQDTSKVFPFYRNKPIDKVEIVRHREYSDIIYEMIFFSNSSEVFRYSSNSTIENNYKRTDSYMIPKQTWKNEEYGHTLSYIKTDNYIFSVVRERRRVAFSWTHTSVDFQNTIDLDNITQIHALKALKVSSDSKIVKGPGHTGGDLVILKDSMDFRVRFLKNVSRQYQVRIRYATNAPKTTVFLTGIDTISVELPSTTSRQNPNATDLTYADFGYVTFPRTVPNKTFEGEDTLLMTLY GTPNHSYNIYIDKIEFIPITQSVLDYTEKQNIEKTQKIVNDLFVN

1. >**BinA** gi|112297518|gb|ABI15165.1| **binary toxin A [Lysinibacillus sphaericus]**

MRNLDFIDSFIPTEGKYIRVMDFYNSEYPFCIHAPSAPNGDIMTEICNRENNQYFIFFPTDDGRVIIANRHNGSVFTGEATSVVSDIYTGSPLQFFREVKRTMATYYLAIQNPESATDVRALETHSHELPSRLYYTNNIENNSNILISNKEQIYLTLPSLPENEQYPKTPVLSGIDDIGPNQSEKSIIGSTLIPCIMVSDFISLGERMKTTPYYYVKHTQYWQSMWSALFPPGSKETKTEKSGITDTSQISMTDGINVSIGADFGLRFGNKTFGIKGGFTYDTKTQITNTSQLLIETTYTREYTNTENFPVRYTGYVLASEFTLHRSDGTQVNTIPWVALNDNYTTIARY PHFASEPLLGNTKIITDDQN

1. >**Cry25Aa** gi|3668335|gb|AAC61892.1| insecticidal protein Jeg74 [Bacillus thuringiensis serovar jegathesan]

MNPYQNKSECEILNAPLNNINMPNRYPFANDPNAVMKNGNYKDWLNECDGITPSIFGTLGVLASIVISTINLATSPSIGDAFALVSSIGEYWPETKTSFPLSVADVNRLIREALDQNAINRATGKFNGLMDTYNTVYLKNLQDWYDTRIPANPQGDSQLREAARRSLEEIERDFRKALAGEFAEAGSQIVLLPIYAQAANIHLLILKDAMQFRTDLGLIRPVGVPITTSAEDPFESEFLLRIKKYTDHCISYYDDGLAKIRSRGSDGETWWEFNKFRREMTLTVLDLVALYPTHNIKLYPIPTQTELSRVVYTDPVGCFGNRKSDIFSRLNFDYLENRLTRPREPFNYLNSVQLFASTVSNSNNGEVLRGNLNKIMFEGGWTASRSGDGVTTGTPFSTMDWSYGWGYPRKHYAEITSRSQALPGLNNSIHVIVGIDSFRAIGPGGQGDHTFSLPGGDMYDCGKVQINPLEDYRNSDHWISDMMTINQSVQLASNPTQTFAFSALSLGWHHSSAGNRNVYVYDKITQIPATKTVREHPMIKGPGFTGGDLADLSSNSDILQYDLRSDYDDRLTEDVPFRIRIRCASIGVSTISVDNWGSSSPQVTVASTAASLDTLKYESFQYVSIPGNYYFDSAPRIRLLRQPGRLLVDRIEIIPVNFFPLSEQENKSVDSLFIN

1. >**cry64Aa** gi|294661779|dbj|BAJ05397.1| crystal protein [Bacillus thuringiensis]

MAIFDVEADLIDNNKWYAQKYYNANPSTFRNPIVYDMNVSDLDVVPITTEFSSTPQLTNSATQVVRNNTSKDQSQTVLFSEKSIETFSRSTTEGYKIGSSIKSTTSFKVKVGFLVSGEINQSIEVAITGEYNHSSTETTTTTNEKLWQVTQPVIIPPYTQVTATLQIFSGPFVVPAKTKATIQGKGTNNGAYNFASAITYTDNSGRVYTDRNRAQALYTDRNEWPGYKRIYVGGSSSTDPTGLLRLEGEARITAQVGLYAVTEFRESPLPGYAGVGSNRTYYAPNILLGDGSVIQFPEYQRYLQR

1. >**Cry53Aa** gi|157418804|gb|ABV55105.1| Cry toxin [Bacillus thuringiensis]

MNSYQNKNEYEILDASQNNSTMSNHYPRYPLAKDPLASMQNTNYKDWLNLCDTPNMENPEFQSVGRSALSILINLSSKILSLLGIPFAAQIGQLWSYTLNLLWPVANNATQWDIFMRTIEDLINVRIETSVRNRALAELEGLGNILEDYKVALRRWDLNPTNLDRQSEVVSQFEIVHAFFRVQMPVFAIRDFEVPLLPVYASAANLHLLLLRDVVINGDRWGLSAARINDYHDLQLRLTSTYTDHCVNWYNTGLNRLIGTNARQWVTYNQFRREMTISVLDIISLFSNYDARRYPTKTQSELTRMIYTDPIGAVGTIGLNPGWLDNAPSFSVIENSVVQSPRTFLFLERVGIFTGVLHGWSSQSQFWSAHRLFLSNLSSIWESIIYGNPQNNIGYEEVDFTNFDVFSINSRATSIMSPFGGGELFGVPRVTFDLSNRTNNSLAQRTYNRPFTFGGQDIVSRLPGETTEIPNSSNFSHRLAYISSFRVGIAGSVLSYGWTHHSVDRHMRLNPNMITQIPAVKXVSGHIVSGPGHTGGDILRVHSGSQGTIIIQSNSAQRYRLRLRYSSTLPGDLILNHRGTDGSQQFIEFTLPATSGQLRFADFTYADGRTVFQTPNSHVFYTVHVQTRSNGIFFIDKIDYIPENTPPLECGGERNLEKEKKAVN

DLFTN

1. >**Cry26Aa** gi|4583416|gb|AAD25075.1|AF122897_1 Cry26Aa1 protein [Bacillus thuringiensis serovar finitimus]

MNSEEMNHVNPFEISDNNDVSIPSQRYPFANDPADSVFCADDFLQSYGEFNMDNFGESEPFIDASGAINAAIGVTGTVLGFLGVPFAGALTTFYQKLFGFLFPNNNTKQWEEFMKQVEALIDEKISDAVRNKAISELQGLVNNITLYTEALEEWLENKENPAVRDRVLQRWRILDGFFEQQMPSFAVKGFEVLLLVVYTQAANLHLLSLRDAYIYGAEWGLTPTNIDQNHTRLLRHSAEYTDHCVNWYNTGLKQLENSDAKSWFQYNRFRREMTLSVLDVIALFPAYDVKMYPIPTNFQLTREVYTDVIGKIGRNDSDHWYSANAPSFSNLESTLIRTPHVVDYIKKLKIFYATVDYYGIYGRSGKWVGHIITSATSANTTETRNYGTIVNHDSVELNFEGKNIYKTGSLPQGVPPYQIGYVTPIYFITRAVNFFTVSGSKTSVEKYYSKKDRYYSEGLPEEQGVFSTEQLPPNSIAEPEHIAYSHRLCHVTFISVSNGNKYSKDLPLFSWTHSSVDFDNYVYPTKITQLPATKGYNVSIVKEPGFIGGDIGKNNGQILGKYKVNVEDVSQKYRFRVRYATETEGELGIKIDGRTVNLYQYKKTKAPGDPLTYKAFDYLSFSTPVKFNNASSTIELFLQNKTSGTFYLAGIEIIPVKSNYEEELTLEEAKKAVSSLFTDARNALKIDVTDYQIDQAANLVECISGDLYAKEKIVLLRAVKFAKQLSQSQNLLSDPEFNNVNRENSWTASTSVAIIEGDPLYKGRAVQLSSARDENFPTYLYQKIDESTLKPYTRYQLRGFVEGSENLDVYLIRYGAAHVRMNVPYNLEIIDTSSPVNPCEEVDGLSHRSCNVFDRCKQSISVAPDANTGPDQIDGDPHAFSFHIDTGTVDSTENLGIWVAFKISELDGSAIFGNLELIEVGPLSGEALAQVQRKEEKWKQVLAKKRETTAQTVCSGEASQLTNSSQILKIRNYDLIQNFRIFSLRNTLSIKFKIYTITNYPYSRLNYDLFMELENRIQNASLYMTSNILQNGGFKSDVTSWETTANAEVQQIDGASVLVLSNWNASVAQSVNVQNDHGYVLRVTAKKEGIGNGYVTILDCANHIDTLTFSACRSDSDTSSNELTAYVTKTLEIFPDTEQIRIEIGETEGMFYVESVELIRMEN

1. >**Cry49Ab** gi|156711540|emb|CAJ86542.1| Cry49Aa protein [Lysinibacillus sphaericus]

MENQIKEEFNKNNHGIPSDCSCIKEGDDYNSLTEVPSEINAKEFSYCSPNMFNLNLPEQSTRFQTIGSIHSNNCSFEILNNDPGYIYGDSVAGECRIAVAHRELGNGLERTGDDRFLFIFYALDNNNFIIANRHDGFVLQFLIANGQGVIVSREYQPNIRQEFTIQSINSDTFRLHSRDTNTFATVCWAQFNSWTKIVSRVDNPGAPNADLRHRSFLDINMPQLPSLTPLQPLPRLTGLEDGGLSPAQAPRAIIGRTLIPCLFVNDPVLRLESRIKQSPYYVLEHRQYWHRLWTDIFNAGERREYREVTGINHNAQNDMNNMINITIGSDGPNRLLFGHLSTPFRQQIISNSNTLGSFANSNYSSRTESITYLNTEFHQVRFARFVKAYEYRLTRADGTLVGTPWVVLDRKEMDLRTFPHNMTLNLENVKIVNADNSYDLSVWKTPLKLKDGKIIIENHENSKPYYN

1. >**Mtx2** gi|1378030|gb|AAC44120.1| **Mtx2 [Lysinibacillus sphaericus]**

MKRTKLLFYIMIASFLFVNGSIYTAKATTIDENNHDIIKQQGVSIEDIDRKIDNMIASIPPLFGFLPYSRFPYIFGESVDVSGINIENTNVTSVVPLFIGSNTFENTTDRTMTFNTVSFSKSITDSTTTQTLNGFKTAFEASGKVGIPLVAEGQIKTTLEYNFSHTNSNTKSVTTTYTVPPQPIPVPPHTKTRTDVYLNQVSISGNVEIYADAITGIKAESSGTVISIGDGLNLASNTFGLIRSPQDPDRVRAIGSGKFNLIHGADFTAITYDITSGEASARIIDVKEISFK

1. >**cry20Aa** gi|2228580|gb|AAB93476.1| mosquitocidal toxin [Bacillus thuringiensis]

MNPYQNNDEIVDVPENYDNNLNRYPYANDPNVAMQNTNYKDWMNGYEEINPSSITAILASIGILNRVIALTGVLGNTQEVISIIQDALGFIRNGTGNELLIHVEQLIQQTLATQYRSAATGAIYGISRSYDNYLMFFRQWERNRTRENGQQVESAFTTINTLCINALAPQASLSRRGFETLLLPNYAMAANFHLLLLRDAVLYRNQWLSNSISTANVNLNILRAAINEYITHCTRWYQDGLNRFDRSSRANMNEWRRFNAYRRDMTLSVLDFATVFPTYDPVLFPAATNVELTRVVYTDPIVMAGGRTAIPGFTRMENLVNSASRVSFLNQMNIYTSFYFRPHNIPRYYWSGNQNFLSNGTSNLYGYRSDGRTTFNVSNIDIFRVNMTTHIGGAFTDDYRGLHRAEFIGANTQNNQRTSLLYSVEIPSSHFRFENHTVFLPGESGLEPNERNYTHRLFQMMNEVSVNPNARGRVFLHAWTHRSLRRTNGLRSDQILQIPAVKTISNGGDRAVVLNYGENIMKLDNLTTGLSYKLTAVDSEASNTRFIVRVRYASMNNNKLNLVLNGAQIASLNVEHTVQRGGSLTDLQYGNFKYATFAGNFKMGSQSILGIFKEIPNIDFVLDKIELIPSNFMSSLEQTQNYNTYNQDTIYTHNQGYDTYDQNSSGMYHQSYNNYDQNMDTTYQPSYDNYNQNASGTYDDGYNPNASDSYDQSYTNNYSQNTNSMYDQGYYNNNYDQHSGCTCNQGYDNNYLK

1. >**cry73Aa** gi|335987509|gb|AEH76822.1| Cry41Aa1-like protein [Bacillus thuringiensis]

MNQNYNHNNYEILDNGNRDYQPRYPLAQAPCSELQNMNYQDVMNTSIGREYQAVQQVNVGEAVSAALGILTTILKAANPTLGAAAGVISSIFGFLWKRFGTDPQAQWKQFMDAVEYLVNQKITDAVRSKAVSELEGVQRALELYQEAADDWNMSPNDADAKERIRRQFTSTNTVIEYAMPSFRVDGFEVPLLTTFAQAANLHLLLLRDAVKFGAGWGLPSAEVEDNYTRLQARTAEYTNHCTNTYDKGLKQAYDLAPNPTDYNKYPYLNPHSKDPIYGKYYTAPVDWNLFNDFRRDMTLMVLDIVAVWPTYNPRLYNNPYGVQIQLSREVYSTVYGRGGSTNSTVDAIESTLVRPPHLVTELTKLTFDERNLYEAETVPVAFKKVTNTLRNVGSSTTWEQSFSSTGGSLKTVHNVWATDIGNLSLNLGAVPLGFSFYNENDQHLTTVGYSGGLWNGIPKGEGSNQNSHHLSYVAALETQSTAGWWPYTYPVNLLGEWGFGWLHNSLTLDNRIATDKITQIPAVKAFKLTPYNNNTKVIKGPGSTGGDLIQLASGFLQYSFPSTDNRQYRIRIRYASADGSALRLRQWIGNGVSDSTHNLPKTYSSGPLTYNTFAIFDTGRIMQTTSSFELWLDNIGSGTLIIDKIEFIPINPIESPPEPKPPVLPGTYQIISALNNSSVVDMDPTSKNVQLYGNGNANNQKWELVFDPSQGAYQVENLANTSLVLTWQVMQGLNVNAAPNLGNPEQYWIIEDAGNGYVYLRNKKDRNKVLDVDHSGTANGTNIQVWDYNGSNAQKFKLNRLS
