# Supplementary material for: Proteomic and metabolomic approach to rationalize the differential mosquito larvicidal toxicity in Bacillus sp. isolated from the mid‐gut of Culex quinquefasciatus mosquito larvae
Source: Anal Sci Adv. 2020 Oct 12;2(11-12):505–14. doi: 10.1002/ansa.202000081 (PMC10989537; doi:10.1002/ansa.202000081)
Supplement: Supplementary file 4 — Supporting Information [file ANSA-2-505-s002.docx]

| **Sr. No.** | **Protein Id/ Gene Id** | **Protein Name** | **Number of Peptide-Spectrum Matches** | **Summed Unique Peptide Precursor Intensity** | **Protein Sequence Coverage (%)** | **Summed Morpheus Score** |
| --- | --- | --- | --- | --- | --- | --- |
| 1 | [P05519](https://www.uniprot.org/uniprot/P05519) | Cry4Ba | 10 | 13119.30 | 5.81 | 26.20 |
|  | [P16480](https://www.uniprot.org/uniprot/P16480) | Cry4Aa | 5 | 15656.55 | 3.90 | 23.22 |
|  | ACF60501.1 | Cry4Ca | 1 | 1654.81 | 1.87 | 6.04 |
| 2 | V9I0N3 | Vip4 | 7 | 14154.51 | 9.22 | 37.22 |
| 3 | [P56957](https://www.uniprot.org/uniprot/P56957) | Cry 22Aa | 3 | 10087.76 | 7.34 | 17.13 |
|  | CAD43578.1 | Cry22Ba | 2 | 7043.19 | 3.64 | 10.23 |
| 4 | BAI44028.1 | Cry 63Aa | 4 | 9992.91 | 10.62 | 21.15 |
| 5 | [Q939T0](https://www.uniprot.org/uniprot/Q939T0) | Cry 34Ab | 4 | 10568.42 | 34.15 | 21.14 |
| 6 | [Q45733](https://www.uniprot.org/uniprot/Q45733) | Cry 9Ca | 4 | 6242.01 | 5.36 | 19.14 |

**Supplementary data 3**: Results of the toxin proteins identified in customized database from 8553 MS/MS spectra of *Bacillus cereus.*

The unique peptides identified in the proteins of *Bacillus cereus* corresponding to larvicidal toxins are highlighted in the sequences

1. >Cry4Ba gi|40354|emb|CAA30312.1| unnamed protein product [Bacillus thuringiensis]

MNSGYPLANDLQGSMKNTNYKDWLAMCENNQQYGVNPAAINSSSVSTALKVAGAILKFVNPPAGTVLTVL

SAVLPILWPTNTPTPERVWNDFMTNTGNLIDQTVTAYVRTDANAKMTVVKDYLDQYTTKFNTWKREPNNQ

SYRTAVITQFNLTSAKLRETAVYFSNLVGYELLLLPIYAQVANFNLLLIRDGLINAQEWSLARSAGDQLY

NTMVQYTKEYIAHSITWYNKGLDVLRNKSNGQWITFNDYKREMTIQVLDILALFASYDPRRYPADKIDNT

KLSKTEFTREIYTALVESPSSKSIAALEAALTRDVHLFTWLKRVDFWTNTIYQDLRFLSANKIGFSYTNS

SAMQESGIYGSSGFGSNLTHQIQLNSNVYKTSITDTSSPSNRVTKMDFYKIDGTLASYNSNITPTPEGLR

TTFFGFSTNENTPNQPTVNDYTHILSYIKTDVIDYNSNRVSFAWTHKIVDPNNQIYTDAITQVPAVKSNF

LNATAKVIKGPGHTGGDLVALTSNGTLSGRMEIQCKTSIFNDPTRSYGLRIRYAANSPIVLNVSYVLQGV

SRGTTISTESTFSRPNNIIPTDLKYEEFRYKDPFDAIVPMRLSSNQLITIAIQPLNMTSNNQVIIDRIEI

IPITQSVLDETENQNLESEREVVNALFTNDAKDALNIGTTDYDIDQAANLVECISEELYPKEKMLLLDEV

KNAKQLSQSRNVLQNGDFESATLGWTTSDNITIQEDDPIFKGHYLHMSGARDIDGTIFPTYIFQKIDESK

LKPYTRYLVRGFVGSSKDVELVVSRYGEEIDAIMNVPADLNYLYPSTFDCEGSNRCETSAVPANIGNTSD

MLYSCQYDTGKKHVVCQDSHQFSFTIDTGALDTNENIGVWVMFKISSPDGYASLDNLEVIEEGPIDGEAL

SRVKHMEKKWNDQMEAKRSETQQAYDVAKQAIDALFTNVQDEALQFDTTLAQIQYAEYLVQSIPYVYNDW

LSDVPGMNYDIYVELDARVAQARYLYDTRNIIKNGDFTQGVMGWHVTGNADVQQIDGVSVLVLSNWSAGV

SQNVHLQHNHGYVLRVIAKKEGPGNGYVTLMDCEENQEKLTFTSCEEGYITKTVDVFPDTDRVRIEIGET

EGSFYIESIELICMNE

>Cry4Aa gi|40352|emb|CAA68485.1| unnamed protein product [Bacillus thuringiensis]

MNPYQNKNEYETLNASQKKLNISNNYTRYPIENSPKQLLQSTNYKDWLNMCQQNQQYGGDFETFIDSGEL

SAYTIVVGTVLTGFGFTTPLGLALIGFGTLIPVLFPAQDQSNTWSDFITQTKNIIKKEIASTYISNANKI

LNRSFNVISTYHNHLKTWENNPNPQNTQDVRTQIQLVHYHFQNVIPELVNSCPPNPSDCDYYNILVLSSY

AQAANLHLTVLNQAVKFEAYLKNNRQFDYLEPLPTAIDYYPVLTKAIEDYTNYCVTTYKKGLNLIKTTPD

SNLDGNINWNTYNTYRTKMTTAVLDVVALFPNYDVGKYPIGVQSELTREIYQVLNFEESPYKYYDFQYQE

DSLTRRPHLFTWLDSLNFYEKAQTTPNNFFTSHYNMFHYTLDNISQKSSVFGNHNVTDKLKSLGLATNIY

IFLLNVISLDNKYLNDYNNISKMDFFITNGTRLLEKELTAGSGQITYDVNKNIFGLPILKRRENQGNPTL

FPTYDNYSHILSFIKSLSIPATYKTQVYTFAWTHSSVDPKNTIYTHLTTQIPAVKANSLGTASKVVQGPG

HTGGDLIDFKDHFKITCQHSNFQQSYFIRIRYASNGSANTRAVINLSIPGVAELGMALNPTFSGTDYTNL

KYKDFQYLEFSNEVKFAPNQNISLVFNRSDVYTNTTVLIDKIEFLPITRSIREDREKQKLETVQQIINTF

YANPIKNTLQSELTDYDIDQAANLVECISEELYPKEKMLLLDEVKNAKQLSQSRNVLQNGDFESATLGWT

TSDNITIQEDDPIFKGHYLHMSGARDIDGTIFPTYIFQKIDESKLKPYTRYLVRGFVGSSKDVELVVSRY

GEEIDAIMNVPADLNYLYPSTFDCEGSNRCETSAVPANIGNTSDMLYSCQYDTGKKHVVCQDSHQFSFTI

DTGALDTNENIGVWVMFKISSPDGYASLDNLEVIEEGPIDGEALSRVKHMEKKWNDQMEAKRSETQQAYD

VAKQAIDALFTNVQDEALQFDTTLAQIQYAEYLVQSIPYVYNDWLSDVPGMNYDIYVELDARVAQARYLY

DIRNIIKNGDFTQGVMGWHVTGNADVQQIDGVSVLVLSNWSAGVSQNVHLQHNHGYVLGVIAKKEGPGNG

YVTLMDWEENQEKLTFTSCEEGYITKTVDVFPDTDRVRIEIGETEGSFYIESIELICMNE

>Cry4Ba gi|40354|emb|CAA30312.1| unnamed protein product [Bacillus thuringiensis]

MNSGYPLANDLQGSMKNTNYKDWLAMCENNQQYGVNPAAINSSSVSTALKVAGAILKFVNPPAGTVLTVL

SAVLPILWPTNTPTPERVWNDFMTNTGNLIDQTVTAYVRTDANAKMTVVKDYLDQYTTKFNTWKREPNNQ

SYRTAVITQFNLTSAKLRETAVYFSNLVGYELLLLPIYAQVANFNLLLIRDGLINAQEWSLARSAGDQLY

NTMVQYTKEYIAHSITWYNKGLDVLRNKSNGQ VVQGPGHTGGDLIDFKDHFKWITFNDYKREMTIQVLDILALFASYDPRRYPADKIDNT

KLSKTEFTREIYTALVESPSSKSIAALEAALTRDVHLFTWLKRVDFWTNTIYQDLRFLSANKIGFSYTNS

SAMQESGIYGSSGFGSNLTHQIQLNSNVYKTSITDTSSPSNRVTKMDFYKIDGTLASYNSNITPTPEGLR

TTFFGFSTNENTPNQPTVNDYTHILSYIKTDVIDYNSNRVSFAWTHKIVDPNNQIYTDAITQVPAVKSNF

LNATAKVIKGPGHTGGDLVALTSNGTLSGRMEIQCKTSIFNDPTRSYGLRIRYAANSPIVLNVSYVLQGV

SRGTTISTESTFSRPNNIIPTDLKYEEFRYKDPFDAIVPMRLSSNQLITIAIQPLNMTSNNQVIIDRIEI

IPITQSVLDETENQNLESEREVVNALFTNDAKDALNIGTTDYDIDQAANLVECISEELYPKEKMLLLDEV

KNAKQLSQSRNVLQNGDFESATLGWTTSDNITIQEDDPIFKGHYLHMSGARDIDGTIFPTYIFQKIDESK

LKPYTRYLVRGFVGSSKDVELVVSRYGEEIDAIMNVPADLNYLYPSTFDCEGSNRCETSAVPANIGNTSD

MLYSCQYDTGKKHVVCQDSHQFSFTIDTGALDTNENIGVWVMFKISSPDGYASLDNLEVIEEGPIDGEAL

SRVKHMEKKWNDQMEAKRSETQQAYDVAKQAIDALFTNVQDEALQFDTTLAQIQYAEYLVQSIPYVYNDW

LSDVPGMNYDIYVELDARVAQARYLYDTRNIIKNGDFTQGVMGWHVTGNADVQQIDGVSVLVLSNWSAGV

SQNVHLQHNHGYVLRVIAKKEGPGNGYVTLMDCEENQEKLTFTSCEEGYITKTVDVFPDTDRVRIEIGET

EGSFYIESIELICMNE

1. >gb|WP_000769760.1|WP_000769760.1 vip4

MKLKSTFKCLTITAVLSQITVYPTTSYAENIDRTINTDKSKEEQNSQGLLGYHFKDNQFEKLSYIEVGIKNKEEEKKQRMKRSIEDEKNLSIQSVRWLGRLVVPETGEYTLSTSFDQHVILQINGETVLNKGKTVKSVSLEKDKAYEVKIEYQNTENIETDLQLFWSINGQDKKLIPHQNIVSPDFSKKENLPEDKLNTALIPNSNLFNGKASSTNMEDTDQDGIPNEWEEKGYTFKNQQIVKWDDSYLSQGYKKYLSNPYKARTIADPYTDFEKVSGHMPAATKEDARDPLVAAYPAVGVGMENLLFSKNENVTEGSSGTMSKSVTDTNTNTNNVDLSAKLGWNDKGFGFEFTPKYSHTWTNSTAVQNSESESWSSQVGINSAESAYLNANVRYYNAGTAPIYDLKPTTNFVLQNSGKSLATITAGPNQIGNSLGPGDTYPKVGQAPISLDKANDAGTVKIPINKDYLDALQSNSEALDLETTQNKGQYGVLDATGQLITDSSKQWDPVRTNIDSVSGSLTLNLGSSKESLERRVAAKNDDDPEDKTPEITIGEAIKKAFNAKEKDGRLYYVNSNGENVFLDESSVNLIGDENTKKDIEQQLEHMEDKKVYNAKWKRGMKITIHVPTSYYDFEKSGDSQWYNTYQDNGGYTGEKTGRINPGSNGYAIKDFTLKPYTSYTARAYVKASSSETDAVFYVDSDINSIGKGIKQNIKATGDKWKLVEMSFNTGSNPELFKKVGFKNQGNVQLQFDDVSVTEWKTEENLEKTHSMENWDVDPSKQYVKGGTFSHVPNSKIRYQWKINDNWEKIIPAPPVDNYGKRVMEKNFNFNDHVELYAVDEHNDYLKVKVAEHNKGDAITEDVLKSSHQFSTWIKSKAPGGGSYTDGWYFERIPDGVLHCVTKYKVSINGGKPVTRDRYNPDKNGRMEVNLLEYNGGRGVKEGSRIEAWAILSNGKEAKVLDKKTS

1. >cry22Aa sp|P56957|C22AA_BACTU Pesticidal crystal protein cry22Aa OS=Bacillus thuringiensis GN=cry22Aa PE=2 SV=1

MKEQNLNKYDEITVQAASDYIDIRPIFQTNGSATFNSNTNITTLTQAINSQAGAIAGKTALDMRHDFTFRADIFLGTKSNGADGIAIAFHRGSIGFVGTKGGGLGILGAPKGIGFELDTYANAPEDEVGDSFGHGAMKGSFPSFPNGYPHAGFVSTDKNSRWLSALAQMQRIAAPNGRWRRLEIRWDARNKELTANLQDLTFNDITVGEKPRTPRTATWRLVNPAFELDQKYTFVIGSATGASNNLHQIGIIEFDAYFTKPTIEANNVNVPVGATFNPKTYPGINLRATDEIDGDLTSKIIVKANNVNTSKTGVYYVTYYVENSYGESDEKTIEVTVFSNPTIIASDVEIEKGESFNPLTDSRVGLSAQDSLGNDITQNVKVKSSNVDTSKPGEYEVVFEVTDSFGGKAEKDFKVTVLGQPSIEANNVELEIDDSLDPLTDAKVGLRAKDSLGNDITKDIKVKFNNVDTSNSGKYEVIFEVTDRFGKKAEKSIEVLVLGEPSIEANDVEVNKGETFEPLTDSRVGLRAKDSLGNDITKDVKIKSSNVDTSKPGEYEVVFEVTDRFGKYVEKTIGVIVPVIDDEWEDGNVNGWKFYAGQDIKLLKDPDKAYKGDYVFYDSRHVAISKTIPLTDLQINTNYEITVYAKAESGDHHLKVTYKKDPAGPEEPPVFNRLISTGTLVEKDYRELKGTFRVTELNKAPLIIVENFGAGYIGGIRIVKIS

>cry22Ba gi|22207625|emb|CAD43578.1| unnamed protein product [Bacillus thuringiensis]

MNSKSIIEKGVQENQYIDIRNICSINGSAKFDPNTNITTLTEAINSQAGAIAGKTALDMRRDFTLVADIYLGSKSSGADGIAIAFHRGSIGFIGTMGGGLGILGAPNGIGFEIDTYWKATSDETGDSFGHGQMNGAHAGFVSTNRNASYLTALAPMQKIPAPNNKWRVLTINWDARNNKLTARLQEKSNDASTSTPSPRYQTWELLNPAFDLNQKYTFIIGSATGAANNKHQIGVTLFEAYFTKPTIEANPVDIELGTAFDPLNHEPIGLKATDEVDGDITKDITVEFNDIDTSKPGAYRVTYKVVNSYGESDEKTIEVVVYTKPTITAHDITIKKDLAFDPLNYEPIGLKATDPIDGDITDKIAVKFNNVDTSKPGKYHVTYKVINSYEKIDEKTIEVTVYTKPSIVAHDVEIKKDTAFDPLNYEPIGLKATDPIDGDITDKITVESNDVDTSKPGAYSVKYKVVNNYEESDEKTIAVTVPVIDDGWENGDPTGWKFFSGETITLEDDEEHALNGKWVFYADKHVAIYKQVELKNNIPYQITVYVKPEDEGTVAHHIVKVSFKSDSAGPESEEVINERLIDAEQIQKGYRKLTSIPFTPTTIVPNKKPVIIVENFLPGWIGGVRIIVEPTK

1. >cry63Aa gi|260268375|dbj|BAI44028.1| M019CP84 [Bacillus thuringiensis]

MSVVYYVKGGDIMDPFSNCSEKKYSDSNNNQELTVESSSFYSNTTNENMKKNYPPINKNFSRNSNDTVLD

ILNISQNNNIDIFAPYNNLHSIKDELQIRTVIPGGVIFSVTGNKFVDSKFTAITYAAITKLTSSLITAAA

TAILGPIGTTIGGAISGPIANALFGLIPGMKPLTPQEIIDIAVEQSKLYTDEQITNLVITNATSELASIK

AKIEDFNSQLNFALNSKNDNLMNRIDFETFLVTLENDIYGSIIKLMNFGYSKQLLPIITVCCTLNLSFLR

DAIFNSQTFNISTQGKRVLTDTFERRTIEYSDKIINEYTLLFNEIKLKENAKTTLDFRTFMSLQVLDQVD

LWSVFKFSQFNIRNTRRLYTIPYQYSENDVSKLDPTQINGDWKFINQILYGLPGNRISGFAGTVELYQPN

KIRRINKLKALYSNNETTGYVGKDANAMDSFDTHPIISQKPAIINYASHVIVNLPPSSVQNLTLLDTIGP

IFPGKYVINQQLYPGLNSLLEYEKFAIPDHKGVNVAGLPNIDSSYTSSTIDNLRQNFITSKPILGSVTAF

QKDIPNYEQVNNKEQIVHLCPTDTDQKLLGFNIPALEYSKDRIANFGFEETWMIIPSYSSSGDNLQFKGT

TAGIKYYLKSAQNAYANYKIFIKIAYKPNNSGNKVQLNINMKDLTSSSIISATLNIQNTSLLKGTSEDNV

KFITFEVPTNFPISNNTYELQLIFTNLQQNDDLRLNELILHPINNDFINILNA

1. >cry34Ab gi|16554919|gb|AAG41671.1| 13.6 kDa insecticidal crystal protein [Bacillus thuringiensis]

MSAREVHIDVNNKTGHTLQLEDKTKLDGGRWRTSPTNVANDQIKTFVAESNGFMTGTEGTIYYSINGEAEISLYFDNPFAGSNKYDGHSNKSQYEIITQGGSGNQSHVTYTIQTTSSRYGHKS

1. >cry9Ca gi|547556|emb|CAA85764.1| unnamed protein product [Bacillus thuringiensis]

MNRNNQNEYEIIDAPHCGCPSDDDVRYPLASDPNAALQNMNYKDYLQMTDEDYTDSYINPSLSISGRDAVQTALTVVGRILGALGVPFSGQIVSFYQFLLNTLWPVNDTAIWEAFMRQVEELVNQQITEFARNQALARLQGLGDSFNVYQRSLQNWLADRNDTRNLSVVRAQFIALDLDFVNAIPLFAVNGQQVPLLSVYAQAVNLHLLLLKDASLFGEGWGFTQGEISTYYDRQLELTAKYTNYCETWYNTGLDRLRGTNTESWLRYHQFRREMTLVVLDVVALFPYYDVRLYPTGSNPQLTREVYTDPIVFNPPANVGLCRRWGTNPYNTFSELENAFIRPPHLFDRLNSLTISSNRFPVSSNFMDYWSGHTLRRSYLNDSAVQEDSYGLITTTRATINPGVDGTNRIESTAVDFRSALIGIYGVNRASFVPGGLFNGTTSPANGGCRDLYDTNDELPPDESTGSSTHRLSHVTFFSFQTNQAGSIANAGSVPTYVWTRRDVDLNNTITPNRITQLPLVKASAPVSGTTVLKGPGFTGGGILRRTTNGTFGTLRVTVNSPLTQQYRLRVRFASTGNFSIRVLRGGVSIGDVRLGSTMNRGQELTYESFFTREFTTTGPFNPPFTFTQAQEILTVNAEGVSTGGEYYIDRIEIVPVNPAREAEEDLEAAKKAVASLFTRTRDGLQVNVTDYQVDQAANLVSCLSDEQYGHDKKMLLEAVRAAKRLSRERNLLQDPDFNTINSTEENGWKASNGVTISEGGPFFKGRALQLASARENYPTYIYQKVDASVLKPYTRYRLDGFVKSSQDLEIDLIHHHKVHLVKNVPDNLVSDTYSDGSCSGINRCDEQHQVDMQLDAEHHPMDCCEAAQTHEFSSYINTGDLNASVDQGIWVVLKVRTTDGYATLGNLELVEVGPLSGESLEREQRDNAKWNAELGRKRAEIDRVYLAAKQAINHLFVDYQDQQLNPEIGLAEINEASNLVESISGVYSDTLLQIPGINYEIYTELSDRLQQASYLYTSRNAVQNGDFNSGLDSWNTTMDASVQQDGNMHFLVLSHWDAQVSQQLRVNPNCKYVLRVTARKVGGGDGYVTIRDGAHHQETLTFNACDYDVNGTYVNDNSYITEEVVFYPETKHMWVEVSESEGSFYIDSIEFIETQE
