# Supplementary material for: Proteomic and metabolomic approach to rationalize the differential mosquito larvicidal toxicity in Bacillus sp. isolated from the mid‐gut of Culex quinquefasciatus mosquito larvae
Source: Anal Sci Adv. 2020 Oct 12;2(11-12):505–14. doi: 10.1002/ansa.202000081 (PMC10989537; doi:10.1002/ansa.202000081)
Supplement: Supplementary file 5 — Supporting Information [file ANSA-2-505-s001.docx]

| **Sr. No.** | **Protein Id/ gene Id** | **Protein Name** | **Number of Peptide-Spectrum Matches** | **Summed Unique Peptide Precursor Intensity** | **Protein Sequence Coverage (%)** | **Summed Morpheus Score** |
| --- | --- | --- | --- | --- | --- | --- |
| 1 | Q03988 | Mtx1 | 9 | 16574.85 | 13.22 | 37.36 |
| 2 | ADV57668.1 | Cry 50Ba | 4 | 4979.60 | 9.13 | 16.08 |
|  | [A0A0F6MTX5](https://www.uniprot.org/uniprot/A0A0F6MTX5) | Cry 50Aa | 2 | 2369.17 | 3.68 | 10.05 |
| 3 | [Q765X5](https://www.uniprot.org/uniprot/Q765X5) | Cry43Ba | 5 | 9285.26 | 6.98 | 28.16 |
| 4 | [B0LUQ8](https://www.uniprot.org/uniprot/B0LUQ8) | BinA | 5 | 5927.31 | 21.89 | 22.30 |
| 5 | [P57092](https://www.uniprot.org/uniprot/P57092) | Cry18Ca | 3 | 4644.53 | 10.36 | 19.07 |
| 6 | [Q45882](https://www.uniprot.org/uniprot/Q45882) | Cry 16Aa | 3 | 5507.37 | 5.71 | 15.05 |
| 7 | [O32307](https://www.uniprot.org/uniprot/O32307) | Cry 19Aa | 2 | 3574.49 | 7.60 | 12.04 |
|  | [O86170](https://www.uniprot.org/uniprot/O86170) | Cry 19Ba | 1 | 4479.84 | 2.79 | 6.02 |
| 8 | [Q93LP5](https://www.uniprot.org/uniprot/Q93LP5) | Cry 34Ba | 2 | 2957.28 | 16.67 | 11.06 |
|  | [Q5GFQ9](https://www.uniprot.org/uniprot/Q5GFQ9) | Cry34Ab | 1 | 2241.39 | 11.38 | 7.02 |
| 9 | ACU24782.1 | Cry60Aa | 2 | 3765.4 | 9.9 | 11.04 |
|  | ADE27985.1 | Cry60Ba | 1 | 1497.45 | 3.13 | 6.03 |
| 10 | [Q0MQM8](https://www.uniprot.org/uniprot/Q0MQM8) | BinB | 2 | 6114.29 | 7.37 | 10.05 |

**Supplementary data 2**: Results of the toxin proteins identified in customized database from 7345 MS/MS spectra of *Bacillus tequilensis*

The unique peptides identified in the proteins of *Bacillus tequilensis* corresponding to larvicidal toxins are highlighted in the sequences

1. >Mtx1 gi|22725708|gb|AAN04906.1| **mosquitocidal toxin [Lysinibacillus sphaericus]**

MAIKKVLKIILAIIIIISCQLPLNQKTVYASPNSPKDNTWIQAASLTWLMDMSSLLYQLISTRIPSFASPNGLHMREQTIDSNTGQIQIDNEHRLLRWDRRPPNDIFLNGFIPRVTNQNLSPVEDTHLLNYLRTNSPSIFVSTTRARYNNLGLEITPWTPHSANNNIIYRYEIFAPGGIDINASFSRNHNPFPNEDEITFPGGIRPEFIRSTYEYHNGEIVRIWINPNFINPSTLNDVSGPSNISKVFWHENHSEGNNMDSKGFILDLDYNQDFDMFAPNGEIPNNNLLNNNSLNVIQNSEYQIKNKKDRNIVVTLDSDYGGSPVESYKNFGFENQKWNIKYDSKKNAYKIYNRETPTLLLSWNSNSSNGEQVIRGYTESGSNNQYWTIEKNVNGFYKFRNLSDPSKILDLKDGNTLNKTPLVVSSENSSSSQEWLIEKTNYQTVKDGTYQVSSKLNENKVIEQISTNKVHIFSNSDKENQVWNLIYNPILKAYKIKSLKYPNYSLAWDSNNTRTIVAATGDYNDQYWLIERNEDNTYIIRNYENRKIVLDLSNGSTTDGNGLLGFEFHGGINQRWIIKPFSFNSIQDGIYQFMTVINQDLIADLTTNNYTIATKTNNYSSNQKWTVTYNDKKRAYKIRNLQHAHLSLAWDSNHSDKIFGATGDYDDQYWIPILQTDGSFIFRNYKNPNKIFGTNGQPINDIPLKAQDVTGQNNQKWYLRHLNSSNNFTGYFNISSKKNFNKIITMNSNKTQAVIFDNIGINNQSWKLKYNDNKNAYQIHILDNFLYFQGGHNIVATMRNVTNDDLRSYWYVEYNFNKDGFIIRNAFDTSYVLDVFQGNFANNTPIITYQNYLNDNQLWNFIPSLGVEPR

1. >cry50Aa gi|89885725|dbj|BAE86999.1| pesticidal crystal protein [Bacillus thuringiensis serovar sotto]

MNLYQNKNEYEILDASRNNSNMSNSYLRYPLESNPNQPLQKTNYKDWVNMCKGNPGGFLLSDEQYVAIVGTVISKLLGFVPVVGDILSFLADTYWPKIAGQEVDTRVWAGLIRHTANLISVENYQDALIKATTNLMGLYGALNVYNRFLADWKKSGMVFNGTLADELRKQMSTLHLMFTQTIIRDFSQPGYQAILLPSYTSAANLHLLLLRDIEIYGKELGFSQQVLDSYYRELILFTKEYTTHCVDTYNAALNAQKQKGWIAFNHYRRNMTLTVLDVITLFPSYDARKYPADKKDVKKLSKTELTREIYTAFLETSPNQTVEIMEASLTRDPHIFTWIKRLDFWTDTLYPDNKFLSANRNGFSYTNSSTVQESIVYGDSGFGSTLTHAIPINSNIYKVSITDTRSIPNRIPQVDFHKMDGTLTSYSSGIKLPPEELRTTFFGFSTNENTPNQPNSSDYTHILTYMKTGIISGGAPKRVSLAWAHKSVNPYNQIFTDDITQVPAVKSSLLNVQAKVIKGPGHTGGDLVALINNNLQAGRMDITCKTSNFNESERRYGLRIRYAANNSFSIYVSYVSSEGSIRQTSRIIESTFSRPNNIIPTDLKYDEFKYNEAFDAILPVRLSPNQSTTISIYQTNALPANQLIIDRIEFIPITQSVLDYTEKQNLEKSQKTVNNLFVS

>Cry50Ba gi|319433507|gb|ADV57668.1| Cry50-like protein [Bacillus thuringiensis]

MNSYQNTNEYEILDASPSYSNMTNSYPRYPLANNRQGSMKNTNYKDWLAMCEGNVEGLFLTDEQMVSIVGAAISKLLGFVPVVGDILSSLADTYWPKIAGQEADTRVWAGLIRHTANLIDNRDVDRVIGQATANVMSLYAALGVYNRFLEQWKSPVKPYAGLADEIRAQMSTLHLLFTTKIISDFTIQGYEAILLPSYANAASLHLLLLRDISIYGEKLGFDSKTLQAYHNEQVKFTTDYTAHCIKTYNLNLNAQKSKGWVAFNQYRRDMTLTVLDLIALFPSYDTHRYPADEKNVKKLSNTELTREIYTALTESSPSKTVEAMEESLTRGPHLFTWPKRLDFWTFNYNMYPDTRYLSANRIGFSYTNSSEIEDSGIYGSPTFGTVLTHQIPLNSNVYRTSITDTTAVPNQVTKMDFYKIDGTNASYNSNITPVPANLRTTFFGFSSDANRPPNQPTVQDYNNILSYIKTDIIGGHQARVSFAWTHKGVNPNNQILTDNVTQVPAVKSSLLNAPARVIKGPGHTGGDLVALLNNGTQAGTMQIQCKTGSFTETSRRYGIRMRYAANNAFTVSLSYTLQGGNPIGITFGTERTFSRTNNIIPTDLKYEEFKYKEYNQIITMNSPQNTIVTIDIRQLNPSSNDQLIIDRIEFIPITQSVLDYTEEQNLETAQAVVDNLFTN

1. >cry43Ba gi|46359602|dbj|BAD15303.1| parasporal crystal protein [Paenibacillus lentimorbus]

MQIIQPSSNALLYSPNKYPYATDPNVIAEGRSYNNWLDTCVGVGDGTRSPEAYAIAEEAVGLSIDILAEI

IYYLGFPIASPLTRALSAIAGQLFSSGDTLMQHIEQLINQKIAEYARNKALAEFQGLGRQYGLYLEALED

WEQNRLSQPHKERVRQTFRILDNSFTSSIPSFAVRNYEVPLLSVYADAANLHLLILRDSYIYGAFWGFDE

DEYYRNYARQIRLSAEYANHCTTWYQTGLRRLQGTRATDWINYNRFRREMTLTVLDICALFSSYDIPSYP

MGTKIQLTREIYTDPVVHSDWLQSTSPGLISFSSLENLVVRAPHLFTWLSRVTIDTGILSTVIGGQYSNN

NFWRTHYQTLRTTGGTSFQSPTYGSTAFPIQRTNTLTFSGDVYTIESSVVTRSSLYGANSVAFTGTTGRS

LYENPTVYPFAQKLIHELPGVDSGRPNATNYSHRLSYISGFSLGYSPSGTGLVYGWTSTTATRENNITLD

DRIVQLPAVKGASLNNCQVVKGTGFTGGDWLKPNNNGTFSMYFAFRSAYTYHFRIRYASSASFSFVISEE

YGRFPTTTVPLLSTMSPLPQNTPFEAFKTVDLPSTVTIRYTSAASTTFQLNFRFTVPGSANVLIDRIEFV

PIEGSLFEYETKQQLEKARKAVNHLFTDGSKKALKEDTTDYEIDQAANVVDCISDECGHEKMILLDEVKY

AKQLSQARNLLLNGNFDDLYPALERENPWKTSPNVTIRQDNPIFKGHYLSMAGANDIEATNDTFPTYVYQ

KIDEAKLKPYTRYKVRGFVGSSKDLELLVTRYNEEVDAILDVPDNIPHAPTPVCGEFDRCKPYSYPPLLP

ECNPEFINQMQPSSCHHNQMVDYNNMNTSTSTTMNPSMNPPLTPEIASSQSGFGRKHRKCHQAHQFEFHI

DTGTIDLVEDLGIWVIFKICATDGYASLDDLEVIEEGALGVEALELVKKREKKWRHQKEQHCSQTKHKYD

AAKHAVMALFTNTRYEKLKFETTISDILYADHLVQSIPYVYNKYVPEVSGMNYELYTELNTLVQNAFYLY

DQRNLIKNGRFSNGLMYWQATPHARVEQEYDRSVLVLPNWDANVSQQLCIEHNRGYVLRVTARKEDPGAG

NVTFSDCANHVDKLSFTSCDIATNAVPGAQANDPAAGVAYGQQGCQIDRVPYGPSGYRADGVAYEQSGHR

TDGVPYRQSGYRADGVAHDQPGYRADGVAYEQSGYRADGVAYEQSGHRADGVPYGQSGYGTDGVTYDQSA

KQTRKYHGCHTDGLPHPEHGCCYPDRVSDGQQLAYVTKSIDLFPDTDKVRIDIGETEGNFRVESVELICM

EK

1. >BinA gi|112297518|gb|ABI15165.1| **binary toxin A [Lysinibacillus sphaericus]**

MRNLDFIDSFIPTEGKYIRVMDFYNSEYPFCIHAPSAPNGDIMTEICNRENNQYFIFFPTDDGRVIIANR

HNGSVFTGEATSVVSDIYTGSPLQFFREVKRTMATYYLAIQNPESATDVRALETHSHELPSRLYYTNNIE

NNSNILISNKEQIYLTLPSLPENEQYPKTPVLSGIDDIGPNQSEKSIIGSTLIPCIMVSDFISLGERMKT

TPYYYVKHTQYWQSMWSALFPPGSKETKTEKSGITDTSQISMTDGINVSIGADFGLRFGNKTFGIKGGFT

YDTKTQITNTSQLLIETTYTREYTNTENFPVRYTGYVLASEFTLHRSDGTQVNTIPWVALNDNYTTIARY

PHFASEPLLGNTKIITDDQN

1. >cry18Ca gi|9622200|gb|AAF89668.1|AF169251_1 parasporal crystal protein Cry18Ca1 [Paenibacillus popilliae ATCC 14706]

MNNYFIGKVLSGHHINNNGNGNTLSRTALTPTNNNVNRGDLVTNGLTPIDNNFIGSNGFIPRNVTRKDPFRKRTTQEFIREWTEWKEKSASLFTAPIVGVITSTLLEALKKLVAGRVLMSLTNLLFPNNSTSTMEEILRATEQYIQEQLDTVTWNRVSQELEGLKNDLRTFNDQIDDFLQNRVGISPLAIIDSINTMQQLFVNRLPQFQVSDDQVLLLPLFAQAVTLHLTFVRDIIINADEWNIPEAQLNTYKRYLKQYVAQYSNYALSTYEEAFRARFYPRNTVENMLEFKTFMTLNVLDLVSMWSLLKYVNLYVSTSANLYNIGDNKVNEGEYSISYWPFFNTYIQTKSNYVLSGVSGYAMRWSYTNPFFGEYIQDHLYNITASYIGGVNGPQIGQQLSTTELDQLVQQQARADIPVDFTQIPINCTLRNPLEVPYYATRFNELTSLGTAGVGGFVRSDVFISNDSVCGLGTNYSSGQTFYPDYYITNISATVQVNGTNTDISPLYFGENRAITSTNGVNKVIAIYNRKTNYDDFTNIRGTIVHEAPTDSTGFTISPLHLDTVNINSYLYIQENYGNNGDSLRVINRAIIKYRLSAARSVIYRLVLRVSGTASSIVAIYENYPVGSANQINTGTDNEGVIDNDSKFIDLIFNTPFSVSGTARELQLQVSGATTSSPLDIMNIILIPINDVPLY

1. >gi|1246432|emb|CAA63860.1| Cry 16Aa cbm71 mosquitocidal toxin [[Clostridium] bifermentans]

MNTNIFSTHLEFSKGVASVFKVIDTIHNISKNNNFNNILTQDFIIDTILSILWEDPNENEIFSSMIEDGE
TITNKNLSAQTKEGLLLNSNSFGLKFKYYNNAFRSWIDNYNPTSIDDVVYRFKDVNSICENNINEFKVKN
YEVTVLPIYMQIANLHLLLLRDGMIYGDAWNLYRELGFSDQDSFYNHVLDKTKFYINDCLNYYNTGLSNL
KLDPNNSWIDITRYCRFMTFYILDMISICPIYDTKVYDKPINMQTLTRKVYSDPVNFIDENIPISEYEKM
YNISPELFSTLFSISFYTNKSGNKFLNGHVNRHVGTDLNYNGLRETHYGNYGSNYEVESMAFDDIKAYSN
NYFNNTQNNNPTSVKSIKFLITKNNDEWIYGEPDSSNIDFTRNIQGYLSNLNNESYTHSLSDMILANNDK
IQINIDTPHSYSYSWIYKGIEDTNYISDKLINQIPLVKEVKLKSRHYSEISVIKGPGFTGGDLILSKVHK
PANQIPAQYMKNKITIPIKTKFPAGSQDFKVRLCYASNHDIGLIRLIAGSKYITTNIQQTFNTTENNPSL
IYDDFKYFNFNETLSITSSGIDELYLEFYYSYTDGNFEDFPKLSIPYTRNYSC

1. >cry19Aa gi|2624006|emb|CAA68876.1| unknown protein [Bacillus thuringiensis]

MLTSGAKNMLKLETTDYEIDQMANAIENMSGEQYSQEKMMQWHDIKYAKQLSQARNLLQNGDFEDLFSGW

TTSNQMSIQADNATFKGNYLHMSGARDIYGTIFPTYIYQKIDESKLKPYTRYLVRGFVGSSKDLELMVMR

YGKEIDTVMNVPNDIPYVPSMPVCNELYDGQQPYPNRHVGYYNPMPVSQPSYTSDTCQCTPGKKHVVCHD

SHQFKFHIDTGEVDYNTNLGIWVLFKISSPDGYATLDNLEVIEEGPVRGEAVTHVKQKEKKWNQQMEKKR

METKRVYDRAKQAVDALFTGEELNYDVTLSHIKNADDLVQSIPYVHNEWLPDFPGMNYDIYQELNARIMQ

ARYLYDARNVITNGDFAQGLQGWHAEGKVEVQQMNGTSVLVLSNWSSGVSQNLHVQHPHGYLLRVSAKKE

GSGKGYVTRMSCNGKQETLTFTSCDGGYMTKTVEVFPESDRVRIEIGETEGSFYIESIELICMNGYTSNN

NQNMSNMYDQSYSGNYSQNTSDMYDQGGSVAKFEKE

>cry19Ba gi|3426160|dbj|BAA32397.1| insecticidal protein [Bacillus thuringiensis]

MNSYQNKNEYEILDAKRNTCHMSNCYPKYPLANDPQMYLRNTHYKDWINMCEEASYASSGPSQLFKVGGS

IVAKILGMIPEVGPLLSWMVSLFWPTIEEKNTVWEDMIKYVANLLKQELTNDTLNRATSNLSGLNESLNI

YNRALAAWKQNKNNFASGELIRSYINDLHILFTRDIQSDFSLGGYETVLLPSYASAANLHLLLLRDVAIY

GKELGYPSTDVEFYYNEQKYYTEKYSNYCVNTYKSGLESKKQIGWSDFNRYRREMTLSVLDIVALFPLYD

TGLYPSKDGKIHVKAELTREIYSDVINDHVYGLMVPYISFEHAESLYTRRPHAFTWLKGFRFVTNSINSW

TFLSGGENRYFLTHGEGTIYNGPFLGQDTEYGGTSSYIDISNNSSIYNLWTKNYEWIYPWTDPVNITKIN

FSITDNSNSSESIYGAERMNKPTVRTDFNFLLNRAGNGPTTYNDYNHILSYMLINGETFGQKRHGYSFAF

THSSVDRYNTIVPDKIVQIPAVKTNLVGANIIKGPGHTGGDLLKLEYERFLSLRIKLIASMTFRIRIRYA

SNISGQMMINIGYQNPTYFNIIPTTSRDYTELKFEDFQLVDTSYIYSGGPSISSNTLWLDNFSNGPVIID

KIEFIPLGITLNQAQGYDTYDQNANGMYHQNYSNSGYNYNQEYNTYYQSYNN

1. >cry34Ab gi|16554919|gb|AAG41671.1| 13.6 kDa insecticidal crystal protein [Bacillus thuringiensis]

MSAREVHIDVNNKTGHTLQLEDKTKLDGGRWRTSPTNVANDQIKTFVAESNGFMTGTEGTIYYSINGEAEISLYFDNPFAGSNKYDGHSNKSQYEIITQGGSGNQSHVTYTIQTTSSRYGHKS

>cry34Ba gi|14571542|gb|AAK64565.1| crystal protein ET80 [Bacillus thuringiensis]

MSAREVHIEIINHTGHTLQMDKRTRLAHGEWIITPVNVPNNSSDLFQAGSDGVLTGVEGIIIYTINGEIEITLHFDNPYAGSNKYSGRSSDDDYKVITEARAEHRANNHDHVTYTVQRNISRYTNKLCSNNS

1. >cry60Aa gi|255653180|gb|ACU24782.1| Cry [Bacillus thuringiensis serovar jegathesan]

MEITDIVLKIYDFIEWDYVTNQDGIPYTLFDKAIYEYELNDTVTIPETKVFKTTPIPIASALTITENRSSQTQLHTIKFSEKKMESVTNTTVHGFKIGGAIKVGAKGTVTANFLVSGGTAEANVELSLTGEYNYSSTTANVNQTEKTWEITENVSVASHTSLTSQLIIMQADIRVPMILNSNLIGKRYYDDYANMFFSYIFQSKTSGRTEMISPASRLANQSWPGKPIVFKSGGSNGSLNLSGFGYSDLYKGLYAFIRYTETPLDRYSSPGKTWDSNLIHLRDGQILNVYDNRGIVKPVRLVE

>cry60Ba gi|292398077|gb|ADE27985.1| crystal protein [Bacillus thuringiensis serovar malayensis]

MTITNIELAIRDYTNWDGTREIPGYINRQVIDGPNIYDYVISDSVAVPKTVIFNVNPTPYTGPNIISENNTDVNQNKRIKFSEKVVETTTHTTTKGFKIGGGIKSTTKGTLKLKFPVGELGFEQTPELPPTGEYNSSSTTGNTCANEKLWEITDNITVPPHSRVTSTLIIMKTEVRVPMELTTNLRGTNSSGEGSFPTSNGLFSYTTSARGTVGGIFVSYYVRPASALYNTSWPDKPATFNSIGSNESLNLLGSGYSDVVPSLYVTIRQDQTPLSGYPGETKTWYSDKVILRDGRIVTLPSNADVNMSQTAKIPYCDRS

1. >BinB gi|166034391|gb|ABY78896.1| **binary toxin B [Lysinibacillus sphaericus]**

MCDSKDNSGVSEKCGKKFTNYPLNTTPTSLNYNLPEISKKFYNLKNKYSRNGYGLSKTEFPSSIENCPSN

EYSIMYDNKDPRFLIRFLLDDGRYIIADRDDGEVFDEAHTYLDNNNHPIISRHYTGEERQKFEQVGSGDY

ITGEQFFQFYTQNKTRVLSNCRALDSRTILLSTAKIFPIYPPASETQLTAFVNSSFYAAAIPQLPQTSLL

ENIPEPTSLDDSGVLPKDAVRAVKGSALLPCIIVHDPNLNNSDKMKFNTYYLLEYKEYWHQLWPQIIPAH

QTVKIQERTGISEVVQNSMIEDLNMYIGADFGMLFYFRSSGFKEQITRGLNRPLSQTTTQLGERVEEMEY

YNSNDLDVRYVKYALAREFTLKRVNGEIVKNWVAVDYRLAGIQSYPNAPITNPLTLTKHTIIRCENSYDG

HIFKTPLIFKNGEVIVKTNEELIPKINQ
